# Clinical Review, and Hospital Reports

**Published:** 1837-07-01

**Authors:** 


### Clinical Review.

### Hospital Medicine and Hospital Surgery.

In Dr. Latham's little volume, intituled Lectures on Subjects connected with Clinical Medicine, there are some observations on physic and surgery, which are not uninteresting.

"At first all students are averse from visiting the sick; they have no fancy for the wards, either medical or surgical, and they especially shrink from the surgical. But when the repugnance is got over, and an interest begins to be felt, that interest is almost sure to be for surgery in preference to medicine; and yet, when they settle in life, their skill in surgery will be little called for, but nine out of ten of the cases which they treat will be medical.

Now, one reason why surgery is more popular than medicine is, that it is easier. Do not, I beseech you, imagine that I wish to disparage surgery. In a profession like ours, nothing can shew such bad feeling, or such bad taste, as purposely to let fall expressions which cast an imputation of inferiority upon those who happen to cultivate a different portion of the same field of science and usefulness from our own. And even here I will allow, if you please, that cases occur in the department of surgery, beset with difficulties and perplexities, which we in the department of medicine do not meet with, and which require information, and judgment, and skill of the highest order to surmount.

But I am now speaking of the ordinary routine of cases, such as we find them in hospitals; and, upon a comparison of such cases, surgery is certainly much easier than medicine; and students take to it the more kindly because it is easier.

Surgery, for the most part, requires fewer circumstances to bring you to a knowledge of its object than medicine does. In surgery there are prominent points of interest, which arrest and command the attention at once; in medicine the points of interest are to be sought after; and, being found, are to be retained and cherished by much labour of the understanding. External sores, external inflammation, and broken bones, require only to be seen and handled in order to be known. But the same knowledge which, in surgery, is obtained by the use of the senses, in medicine, which is conversant with internal disease, can only be acquired by a process of reasoning; and reasoning is more difficult than seeing and touching, and its conclusions are more uncertain, and much more liable to error.

Moreover, the adaptation of curative means requires more vigilance in medicine than in surgery. There is no end of the circumstances to be taken into consideration day after day, in order to practise medicine with tolerable success. A man has an *external* inflammation; the surgeon sees it, and is at once sure of its existence; he prescribes for it, and sees its gradual decline as plainly as he first saw its rise and progress. A man has an *internal* inflammation; but the physician, not seeing it, is obliged to come to the knowledge of its existence by a great variety of considerations: he prescribes for it, and is again obliged to enter into a variety of considerations before he can know that it has begun to decline, or has ceased. The uncertainty of physic I readily admit; but I do not admit the vulgar reproach which has followed from it. There is nothing absolutely sure but what rests upon the basis of numbers, or falls within the sphere of the senses. Where reasoning begins, there begins uncertainty; and on this account the highest and the best things in the world are all uncertain, and so is our profession. But from this very uncertainty those who practise it successfully claim their greatest honour; for where there is no possibility of error, no praise is due to the judgment of what is right.

Another reason why surgery is more popular than medicine is, that it is easier for pupils to make surgical cases a matter of discussion and conversation among themselves, and thus to convey an interest to each other respecting them. They can agree about the extent of this burn and that fracture, and understand each other when they talk about them; but concerning the progress of a fever, and all its circumstances—how they differ to-day from what they were yesterday, and what influence the means employed have had in determining the changes which have taken place—it is quite impossible that they should have any very general conversation. It is necessary to be in the presence of the patient to point them out. Language often fails of terms to designate them; and the most experienced often find a difficulty in making themselves intelligible to each other in speaking of them. There once flourished within these walls 'The Medical and Philosophical Society of St. Bartholomew's Hospital.' I fear it exists no longer. The time was that it was attended weekly by at least an hundred students and others. There was often no lack of discussion, and good discussion too, upon professional subjects; but the subject was almost always a *surgical* subject. I have already shown the reason why it was so. It could not be otherwise.

Again: young men like to be doing something—something that shall be real employment. Thus they are gratified while they are plastering, and binding, and dressing, &c. They see and they feel that they are promoting some object daily and hourly, with their own hands, for the benefit of the sick. But in medicine, the quiet and almost passive manner in which they are engaged about the sick, requires a state of mind which is seldom possessed in early life.

Why do I mention all these things? In order to shew you that I am well aware of all the circumstances which are apt to abate your interest for that department in which it is my duty and my desire to promote your instruction, and of all the difficulties which I have to encounter, when I attempt to win you to it."

No doubt there is much truth in this; but probably there is not the whole truth. Surgery is not merely easier than physic, it is more exact, and, from its exactness, it is more satisfactory.

One man has a pain in his bowels. The physician says that he has inflammation in them. In accordance with his opinion, he prescribes leeches, perhaps calomel and opium, or some other medicines. The patient grows better, gets well, and there is an end of the matter. There is not much of demonstration in a case like this, nor is there much to interest a student independently of demonstration.

Another man has a pulsating tumor in his ham. It is increasing, and the student sees that it is a dangerous, and, if unheeded, will soon be a mortal disease. The surgeon says it is an enlargement of an artery, and explains that if that artery is tied nearer the heart, and the current of blood through the tumor diminished, the pulsation in it will cease, and ultimately the tumor itself will disappear. The surgeon *does* tie the artery in the thigh, the pulsation of the tumor *does* cease, the tumor *is* finally absorbed. In such a case there is much of demonstration, much to arrest and to fix the attention of a student.

There is a "pomp and circumstance" in the practice of surgery, which there is not in that of physic. There is all the solemnity and all the éclat of the capital operations. In an amphitheatre, crowded with spectators, the surgeon is seen plunging with confidence and coolness his formidable instruments into the inmost recesses of the body, and, in the midst of blood and groans, he extracts the calculus, or ties the vessel. All this is adapted to seize the attention of the student, who perceives at once the value of knowledge both to the surgeon and the patient. When he contrasts the physician smelling a stool, with the surgeon wielding the catlin or the gorget, is it surprising that he is caught with the glitter and display of the craft of the latter?

Then there is the novelty of operative surgery. As an apprentice, the pupil has made up pills, and compounded draughts, nay he has seen something, perhaps much, of their employment. Practically, he knows a little of the materia medica, practically, he knows less, but still something, of medicine. But anatomy and physiology, and surgery which hangs by them, are all new things to him. The charm of freshness is added to those of comparative simplicity, and demonstration, and the result is, as Dr. Latham has remarked, a preference for what, a very moderate acquaintance with the human mind, would lead us to suppose would be naturally preferred.

---

### Hull General Infirmary.

Report of some Cases. By Mr. Henry Snowden.

For the succeeding cases we are indebted to the gentleman, whose name we have inserted above. We have not the pleasure of an acquaintance with him, but, if we may judge by the present report, and by his communications to the Medical Gazette, he evinces a zeal for the profession which redounds greatly to his honour.

#### 1. Softening of the Brain.

George Bigsby, ætat. 57, entered the hospital January 7, 1836. Six days previously he was attacked with vertigo, and a dull pain in the head, which he referred to the right parietal region; this recurred only at intervals. At the same time the sensation of the right half of the face became diminished. There was also profuse lachrymation of the right eye. He had enjoyed in general good health, but had been an intemperate liver.

On admission the following symptoms were observed. Face flushed: conjunctivæ injected—protrusion of the globe of the right eye, which deviated to the left side, its vision was much impaired, and the sensibility of its mucous covering abolished. Left commissure of mouth drawn upwards—sensation of the right half of the face diminished. The motion of the left extremities (particularly of the upper) impaired, with a sense of coldness. Vertigo, pulse 85 and full, tongue white, bowels open daily, excretions passed voluntarily, sleep undisturbed. There is no disturbance of intelligence.

Dr. Alderson, under whose care he was admitted, prescribed

V. S. ad ℥xij.

C. C. ad nucham, postea empl. lyttæ ibidem. Capt. Olei. ricini ℥ss. statim—Mistur. salin ℥j. 4tis horis.

16th. Cutaneous sensibility of the left inferior extremity diminished. Torpor of the intelligence. Pulse small. App. empl. lyttæ ponè aurem dextram; curetur ulcus unguent. sabinæ.

22nd. Vision of the right eye lost—muscular debility. He was ordered Infus. rosæ c. ℥j. Magnes. sulph. ʒss. ter in die.

Feb. 1. Improvement as to the motion and sensation of the extremities, other symptoms the same. He was asked repeatedly if he had pain, but always replied in the negative, frequently however he would raise the sound limb to the right side of his head, as if some undefinable sensation were there.

From this time to the 25th, no material change was observed, if any thing there was amendment of the paralysis.

On the 25th, a slight cough with dyspnœa announced the existence of pulmonary disease. Over the inferior lobe of the right lung the stethoscope discovered a crepitous râle. There was no heat of the skin, and the pulse being 78 and small precluded the employment of active treatment.

Daily aggravation of the pulmonic symptoms, orthopnœa, asphyxia, and death on the 7th of March.

*Inspection twelve hours after death. Cranium.*—The subarachnoid cellular tissue infiltrated with serum, and here and there opaque. In the right hemisphere of the cerebrum were two softenings, each of the size of a large nut, one situate in the middle, the other in the posterior lobe. In the middle lobe of the left hemisphere a softening of like extent. In the right hemisphere of the cerebellum a softening of the size of a pea, in the left, one fully equal to a large walnut. The superior portion of the pons variolii was a mere pultaceous mass. These were all red softenings. In the right corpus striatum and right hippocampus major were small points of yellow softening.

*Thorax.* Lungs generally engorged. The parietes of the heart were soft; a tuberculous mass was found in the posterior mediastinum.

*Abdomen.* Kidneys soft, other viscera healthy.

The functional disturbances in this rare case were by no means proportional either in severity or variety to the extent and locality of the softenings, neither were there any symptoms, except those affecting the eyeball, which aided the diagnosis in particular. The circumstance of the patient when questioned as to his suffering pain, applying the sound limb to the side of the head, which sign is considered by Rostan of great value, assisted in the general diagnosis.

### 2. Cases of Adhesion of the Pericardium.

Edward Shields, ætat. 22, was admitted into the Infirmary August 26, 1835, under the care of Dr. Alderson. He complained of pain and tightness over the chest, had frequent palpitations, dyspnœa, slight cough, with copious mucous expectoration. His countenance was pallid and anxious, lips purple, pulse 90, full and intermittent (a pause every third beat), tongue clean, appetite good. He could not endure the recumbent posture. He stated that he had been ill six months, he attributed his complaint to an inflammation of the side. He had had rheumatism.—There was a slight prominence of the cartilages of the præcordial ribs. The motion of the heart was abrupt and unsteady. A loud murmur resembling the crackling of new leather accompanied both sounds.

He was ordered leeches to the region of the heart. Calomel in small doses. He continued in this state till the 4th of September, on which day, after eating a full meal, he died suddenly.

On inspection 24 hours after death, there were found pleuritic adhesions. The pericardium adhered to the heart in the greater part of its extent, in the free spaces were patches of lymph, apparently of recent formation. Heart hypertrophied and dilated.

Mary Perkins, ætat. 20, received into the hospital April 16, 1836, under the care of Dr. Alderson. Two years ago had had rheumatism. She stated that since that time she had been subject to palpitation of the heart, it did not however, produce much inconvenience till about a month before her admission.—She then was attacked with dyspnœa, slight cough, trembling and faintness. These symptoms became more urgent. On admission, in addition to the above, the following symptoms were observed. Countenance pallid, lips of a purple hue, pulse 84, full and thrilling, hands puffy, tongue clean, appetite good, decubitus in any position. Above the clavicles a purring tremor and strong pulsation were sensible to the touch; the impulse of the heart was slightly increased, but long in being effected, the apex struck the thoracic parietes to the left of and inferior to its usual position. The purring tremor was evident in all the arterial trunks, it was traceable also along the sternum to the heart.

She continued in this manner for some days, when suddenly she was attacked with violent vomiting. A few days before death, which occurred on the 21st of May, the ventricles contracted irregularly, and the dyspnœa was excessive.

On inspection, 24 hours after death, the pericardium was found universally adherent to the heart. The heart was greatly dilated, the dilatation being chiefly confined to the left side. The margins of the semilunar aortic valves were thickened.

Charlotte Lenham, ætat. 15, entered the hospital March 2, 1837, under the care of Dr. Alderson. Two years ago had been in the hospital for acute rheumatism, during the progress of which pericarditis supervened. She left the hospital, cured of her articular rheumatism, but her heart laboured, and she had dyspnœa. She was long an out-patient. She re-entered the hospital in October, 1836. After a little time she returned home. When again admitted she presented the following symptoms. Countenance pallid, cellular tissue infiltrated with serum, short *hacking* cough, no expectoration, urgent dyspnœa, decubitus impossible, palpitations, pulse from 90 to 100, full and regular, occasional pains over the region of the heart, sleep disturbed, appetite impaired.

The impulse of the heart slightly increased, the first sound was louder and heard over a greater extent than usual. There was no abnormal murmur.

She was ordered leeches to the region of the heart. Calomel and digitalis in small doses. Acupuncturation of the legs. On the 16th of March violent vomiting set in, this aggravated all the symptoms. On the 21st death.

On inspection there were found pleuritic adhesions. The pericardium was universally adherent to the heart. The heart was dilated and slightly hypertrophied. Pale serum in the peritonæal sac.

### 3. Tubercular Sycosis of the Legs.

Two cases of cutaneous disease of a species hitherto, I think, undescribed, occurred during the Autumn of the past year. In one case a mass of tuberculous or mammillated elevations extended in a serpentine direction from the inner malleolus to the tubercle of the tibia. From these exuded a thin purulent fluid, and here and there the surface was covered with dark incrustation. In the other case the character of the disease was the same, but it was less widely diffused. The disease in both cases had existed several months before the patients entered the hospital, and a variety of applications had been ineffectually employed. Struck with its analogy to sycosis menti, Dr. Alderson suggested the application of a poultice of linseed meal. The poultices were changed thrice daily. In six weeks the disease was cured.

---

### Doncaster Deaf and Dumb Institution.

### Differences in the Power of acquiring the Language of Gesture in the Deaf and Dumb.

The following observations are extracted from the Phrenological Journal for June, 1837. They are communicated to our contemporary by Mr. Scott, of the Deaf and Dumb Institution.

"The uneducated deaf and dumb have no knowledge of our language, and can only express their thoughts and desires by gesture, as nearly representative of such as they can make them. Thus deprived, in a great measure, from communicating with their fellow beings, or gaining any knowledge from books, the first and great aim in their education, is to give them the power of comprehending and using language as a means of communication. An instructor, under such circumstances, has a good opportunity for observing the different degrees of aptness, if such exist, in acquiring this knowledge, and whether it be always in proportion to the powers exhibited in acquiring other kinds of knowledge. There is also an opportunity of observing whether these powers, and the development of the phrenological organs, be in harmony.

In the case under consideration, the boy, who is about fifteen years of age, has been at school upwards of four years, and the progress he has made in the knowledge of words is extremely limited. One with a moderate capacity for such acquirement would do as much in half the time. But this want of talent is confined to language. In many other branches of knowledge he is decidedly superior to most of the children in the school. He draws correctly and with ease. In calculations of number, he is about on an average with the generality. What, however, he most delights in, is in constructing pieces of mechanism; in this occupation he would be constantly employed, and he is very clever in the use of tools. Any piece of mechanical work he sees, if it be not very complicated, he can comprehend, and imitate. The phrenological development, I think, on examination, you will find in perfect accordance with the character I have shortly given. His perceptive organs are all good, with the exception of Language, which is certainly small. Individuality, Weight, and Size are large, Constructiveness is very large, and in satisfying this propensity is his chief pleasure.

I remarked, that the mode of communication used by the deaf and dumb, when unable to apply language, is that of imitative signs. There is in this often a great difference exhibited by different children, in the minuteness and clearness with which they relate what they wish to express. Some give only an outline, as it were, of the circumstances, the more prominent features; others will not only give the outline, but fill up the detail, with striking exactness, putting in every little incident which can illustrate or give character to the representative."

---

### HOSPITAL OF THE 2nd DRAG. GUARDS.

### SUPPOSED GLANDERS IN THE HUMAN SUBJECT.

Mr. Brown, surgeon of the 2nd Dragoon Guards, has published the following case in our contemporary of Dublin.* The case occurred in the Hospital of the Dragoon Guards, at Cahir Barracks, in Ireland.

*Case.* Corporal John Wells, æt. 38, a strong and healthy man, was suddenly awakened from an unrefreshing sleep, on the 16th of April last, by rigors, headach, and irritability of stomach, all of which continued unabated when admitted into hospital next morning; complaining, in addition, of severe continued pains, and stiffness in all his large joints, which became excessively aggravated on the slightest motion. It appeared that he had had sole charge, for some time, of a glandered horse, which had been destroyed on the evening of his attack, and that he had exerted himself greatly in cutting up and burying the carcass. Though the symptoms he displayed are the constant precursors of a combination of severe acute glanders, and farcy in the horse, no suspicion of the sort was entertained with respect to him. He was treated for acute rheumatism, but on the morning of the 19th, he was so much worse that Mr. Brown and Dr. Home became alarmed.

"From this period, the constant and general pain, night and day, became excessive, and violent to a degree; but particularly over the left shoulder, which, on examination, shewed the scapula slightly tumefied, although not inflamed; but being above the temperature of health, leeches were frequently applied over its entire surface; and it bled profusely for some hours after, without affording the least relief; but it shortly after became hard, ecchymosed, and insensible to the touch.

The severity of his sufferings continued unabated; and on the morning of the 24th, seven days after admission, the tumour over the scapula had assumed a dark livid colour, and attained a considerable size, resembling, in a strong degree, the shoulder of a man recently and severely punished.

Similar tumefactions, but more circumscribed, were now observed on the legs, arms, and sacrum, and one of considerable magnitude over the left temple, which had already distorted the entire face. The eye being apparently diminished and humoury, the lids tumefied, the inferior one with a prominent doubling in it, the conjunctiva pale and infiltrated, as well as the membrana nictitans and caruncula lachry-

* Dublin Journ. May, 1837.

malis. The skin and cellular membrane of *this* tumor, together with *those* on the extremities, became like *that* on the scapula, hard, insensible to the touch, and of a dark chocolate colour: convincing us that the application of leeches to the original one, was not instrumental in the production of those appearances, as we had then supposed. The right nostril was likewise contracted, and gummed with an inspissated discharge; and he complained of constriction of the throat, with difficulty in swallowing cold liquors, but not those previously warmed; and on examination, the posterior fauces were found much inflamed, and nearly of the same purple hue as the *tumors* on the surface; the whole of which observed regular gradations from their commencement: first showing themselves, not simultaneously, but in succession, by a slight discoloured puffiness of the skin and cellular membrane; generally nearest the bone; they were next observed after a lapse of twelve or fifteen hours, diffused over their entire surface with a deep vermilion blush, which then changed rapidly into a dark brown; the integuments becoming thick and callous, with fissures or superficial cracks, from which exuded a thin, acrid, corrosive sanies. These formed their characteristic appearances without any very material deviation, or producing the slightest mitigation of suffering throughout, which had now become so excruciating as to baffle every effort of art to procure either sleep or rest: not even whilst in the warm bath has he had a moment's respite from pain.

His thirst from the beginning had been great, with a foul parched tongue; his pulse varying from 88 to 96, and full, but easily compressed; and the blood abstracted at the commencement of this disease appeared much attenuated, buffed, and deprived of the coagulating principle. His bowels (constantly attended to) were easily kept free, and his excretions, both urinary and alvine, were always natural in every respect, showing the alimentary canal perfectly healthy.

In this state he advanced into the morning of the 28th, his eleventh day under treatment; when several distinct, warty pustules, considerably raised above the surface of the skin, were first observed on different parts of the body, very much resembling yaws, but particularly numerous and large over the right side of the neck and shoulders, and on the inside of the arms and thighs."

The patient survived, in a state of partial somnolency and low delirium, until the 30th, when he died.

*Dissection.*—"The entire surface exhibited a most unsightly deformity, with extreme emaciation, being nearly covered by black gangrenous tumors of various sizes, each surrounded by numerous small vesications about the size of peas, which, with those over the neck, shoulder, arms, and thighs, at first sight resembled the yaw pustule, but on cutting into them they were found to be merely elevations of the cuticle filled with a dark, violet-coloured, inspissated lymph. Glanders being suspected, the fingers, &c. were searched for a scratch, but none was found, nor were the absorbents discovered to be inflamed. Beneath the scalp, over the left superciliary ridge, was a cluster of tuberculated bodies, imbedded in the cellular tissue, external to the pericranium.

"At this stage of the dissection, the presence of our highly talented and much esteemed veterinary surgeon, Mr. Woodman, was solicited, and on his arrival he unhesitatingly recognized a strong resemblance between *these* and *those* usually found in the nasal linings of glandered horses after death."

The mucous membrane lining the frontal sinuses and æthmoidal cells appeared throughout not only pale, thickened, and infiltrated; but in the right frontal sinus was found another cluster of what Mr. Woodman considered to be "well-defined, ulcerated tubercles, and exactly similar in appearance to what we have in the membrane lining the frontal sinuses and other cavities of the head in *acute* glanders in the horse."

The posterior fauces were of a dark purple colour, and on the right tonsil there were four or five ulcerated patches of a similar character with the preceding.

"We now resumed the examination of the trunk, first inspecting a large, hard, cancer-like tumour, spreading backward over the scapular region, and downwards by the serratus and latissimus dorsi muscles, the most prominent part having cracked or separated previous to death, from which exuded a thin, highly fetid ichorous sanies; and on cutting through this disorganized mass down to the bone, the muscles appeared perfectly decomposed, and of a dark liver colour, (exhaling a peculiarly fetid odour, similar to cariosity,) with points of purulent matter, as it were, infiltrated everywhere through its entire substance, resembling much a hepatized or tuberculated lung; and on removing the whole of this diseased mass from the bone, the scapula was observed nearly covered by a cluster of grey, circular tubercles, the whole composed of fine cellular tissue, enveloped in small cysts, and firmly attached to the periosteum, differing only in this respect from those found in the pericranium."

The other tumors of the sacrum and extremities had all characters exactly analogous to the preceding.

The muscles, generally, were blanched and flabby, the fibres softened, and the cellular membrane infiltrated with a yellow serosity.

Mr. Brown observes that in this case there was no *evidence* of inoculation of glanders. Yet the phenomena render it not unlikely that the patient had imbibed in some measure that poison. At the same time, we must view cases of this nature with extreme suspicion, and must be slow to admit of specific infection unless the proof is extremely strong.

Modern investigations have shewn that the affections of the cellular membrane are so various, so severe, and occasionally so complicated, and the phenomena connected with the re-absorption of pus are so startling, that the best-informed pathologists will be the last to extend implicit credence to reputed specific inoculations or contaminations. We cannot say that we have seen a case exactly like the preceding, but certainly we have seen many cases of diffuse cellular inflammation and purulent deposites which might readily be conjured up into instances of poisoned wounds. We do not make these observations with the view of throwing cold water on the present case, but simply for the purpose of recommending caution and rational scepticism.

---

### NORTH LONDON HOSPITAL.

### DISLOCATION OF THE CARPUS BACKWARDS.

Hannah Hussey, aged 15, admitted April 22d, 1837. She stated that her right hand was resting on the end of the heavy box of a mangling machine, whilst her brother was at the wheel by means of which the box is moved upon rollers; the latter gradually approached a wall behind her, and "*jammed*" the forearm lengthways between the machine and the wall, producing the dislocation. She immediately came to the hospital, arriving there about a quarter of an hour after the accident, when the nature of the injury received was very evident. She suffered great pain;—the forearm and hand were in the prone position, and presented a very distorted appearance;—the forearm was shortened;—there was a remarkable prominence on the back of the radius and ulna, the anterior surface of the first row of carpal bones lying upon the posterior surface of the ends of the radius and ulna;—above this prominence was a deep depression, and the projection presented an arched appearance, the convexity towards the elbow;—the ends of the radius and ulna were very apparent on the palmar surface, and the styloid processes were easily distinguished;—below these, there was another deep depression. The fingers were not rigid—they were extended, but not violently so, being supported in the hand of her mother as she came to the hospital; she had not the least power of moving them, or of supining her arm. With the assistance of Mr. Burton, I immediately reduced the dislocation; he grasped the fore-arm, immediately below the elbow, with both hands. I grasped her hand with my right hand, and the wrist with my left, placing my thumb over the dislocated carpal bones. By means of firm extension with my right hand, and pressure with the thumb, the bones were readily returned to their natural situation—gradually and without any noise. After the reduction the wrist was exceedingly moveable:—there was then no swelling, and it was easily ascertained that there was no crepitus, and that the moveability did not depend upon fracture of the radius.

The fore-arm was placed in a sling; she was directed to foment the wrist frequently with hot water; and some aperient medicine was given to her.

April 23rd. This morning I proceeded to her residence (No. 9, Little Edward Street, Cumberland Haymarket), for the purpose of ascertaining exactly the manner in which the injury had been produced. A mangling machine is placed along one side of a room, and the frame-work is within three or four feet of a wall at right angles with the other one, in order to allow of the movement backwards and forwards, of the heavy box beyond the frame-work. The box contains a ton and a quarter of iron, and is moved upon rollers. Her brother was gently turning the handle, and the girl's right hand was placed upon the end of the box, when her attention was directed to a dog which she was fearful would frighten some chickens which were in the room; the box gradually approaching the wall, the elbow reached it, and the carpus was dislocated backwards. Her brother reversed the direction of the box, immediately that she hallooed "stop." No laceration or bruise is observed on the elbow.

Mr. Liston examined the arm. There is now great effusion in and around the joint, extending to about two inches above it on the palmar, not quite so high on the dorsal surface of the arm; there is considerable redness of the integuments. She cannot extend the fingers in the least; but can flex them slightly. She was directed to continue the fomentation; the bowels have been freely opened.

April 25th. The swelling and redness are still extensive. She can extend the fingers very slightly, which is attended with pain along the back of the arm; and flexion is attended with great pain along the fore-part of the arm. Continue the fomentation.

April 27th. The swelling and redness have somewhat diminished. To-day Mr. Liston again very carefully examined the wrist, and came to the conclusion that the radius was not broken.

The preceding case has been communicated to us by Mr. Herbert Barker, the zealous and intelligent house surgeon of this hospital. We present it without any alteration.

---

### Guy's Hospital Reports. No. IV. for April, 1837.

The present number contains few articles, but much matter. Insulated cases are almost banished from its pages, which are devoted either to original essays or to general results—a judicious, and, as probably, the event will prove, a successful plan.

The contents of the number are as follows:—

1, A Practical View of Lithotrity, with Remarks on the Lateral Operation of Lithotomy; by Mr. Aston Key—2, Observations on the Diagnosis of Pneumonia; by Dr. Addison—3, Two Cases of Fatal Poisoning by Arsenious Acid, with Remarks on the Solubility of that Poison, in Water and other Menstrua; by Mr. Alfred S. Taylor—4, An Essay on the Safety-valve Function of the Right Ventricle of the Human Heart, and on the Gradations of this Function in the Circulation of Warm-blooded Animals; by Mr. T. W. King—5, An Experimental Inquiry respecting the Process of Reparation after Simple Fracture of Bones; by Mr. Bransby Cooper—6, Summary of Cases in the Obstetric Ward—Statistical Account of the Lying-in Charity—Reports of Obstetric Cases; by Dr. Ashwell—7, Description of a Remarkable Specimen of Urinary Calculus, to which are added some Remarks on the Structure and Form of Urinary Calculi; by Dr. Hodgkin—8, Cases and Observations illustrative of Diagnosis, where Tumors are situated at the Basis of the Brain; or where other parts of the Brain and Spinal Cord suffer Lesion from Disease; by Dr. Bright—9, Illustrations of the Museum—by Dr. Hodgkin.

These nine articles are spread through 318 pages, and their length, must, therefore, be considerable. Indeed it is very creditable to the officers of this hospital that they are able and willing to furnish the materials, at short intervals, of volumes at once so bulky and so valuable as these Reports have hitherto been. The first paper of which we shall give an account, is:—

### 1. A PRACTICAL VIEW OF LITHOTRITY; WITH REMARKS ON THE LATERAL OPERATION OF LITHOTOMY. By MR. ASTON KEY.

Mr. Key commences with some candid, and, as it seems to us, judicious observations on the relative value of lithotrity and lithotomy. He dwells on the exaggerated estimate which some from enthusiasm, or, from other motives, have advanced, and which others from credulity have implicitly received, of the merits of the former. It has been represented as an easy, a painless, a safe, and a successful operation, when, too frequently, it is neither easy, painless, safe, nor successful. Mr. Key's sentiments coincide to the letter with those which we expressed in the 50th Number of this Journal.* We need not, therefore, repeat them, but content ourselves with relating the more prominent facts brought forward by Mr. Key, facts which deserve attention because he personally vouches for their authenticity.

Mr. Key relates twelve cases. The first we shall allude to are those in which the operation of lithotrity was successful.

*Case* 1. A Mr. L. was sounded by Mr. Key, who discovered a stone of a size well adapted for lithotrity. As his bladder was not irritable, it was decided that a well-known lithotritist should operate upon him. The operation was performed at several sittings, and with complete success.

*Case* 2. "Mr. C——, a surgeon, was brought to me by Dr. Gordon, in order to ascertain if he had stone in the bladder; as certain symptoms, of which he complained, were considered to depend on the presence of a calculus. On sounding him, I struck a small calculus; and he expressed a desire to undergo the operation of lithotrity, under the same operator. After one stone was broken down, a second was discovered; and he underwent several operations; the operator telling him that he had crushed several entire calculi. Mr. C—— returned home, assured that he was freed from the cause of his disorder. I saw him in three weeks after the last operation, and his bladder still continued to be irritable; but this symptom gradually ceased; and he appeared, when I last saw him, to be altogether freed from his disorder."

*Case* 3. "A surgeon in active practice, was seized with considerable hæmaturia after exercise, and soon complained of symptoms that gradually assumed the character of vesical calculus. On sounding him, a small stone was discovered; and he placed himself under the care of a lithotritist. The stone was crushed after three operations, and he obtained entire relief from his disorder. He now remains well.

These are all the successful cases which Mr. Key can report. The next, six in number, are those in which lithotrity proved unsuccessful.

*Case* 4. "In 1829, an Irish gentle-

---

* *Art. Lithotomy and Lithotrity. Pp.* 465, *et seq.*

man, Mr. H——, about 64 years of age, arrived in this country from Paris, to obtain professional advice for a vesical disorder. I learnt that he had undergone the operation of lithotrity under M. Civiale, about six weeks previously; and that the operation had been followed by acute inflammation of the bladder, for which M. Amusat was consulted. As soon as the cystitis subsided, he came over to this country, labouring under symptoms of stone in the bladder, which, in severity, I have only on one occasion seen exceeded.—The desire to void urine was nearly incessant, and the attempt excruciating, and often unsuccessful. He lingered out a miserable existence for a few weeks, receiving such relief as medicine could afford. On passing a flexible catheter to relieve the bladder, I discovered a considerable fragment of stone. Sir Astley Cooper saw this gentleman several times, and considered him beyond the reach of any operation. He died with his sufferings unmitigated."

*Case* 5. "Mr. E——, between 50 and 60 years of age, had been annoyed for some time with symptoms of disorder, which Mr. Giraud, of Feversham, found to arise from a calculus in the bladder. He came to town, at the request of Mr. Giraud, to consult me. Finding the stone small, I gave him the alternative of the two operations. He preferred lithotrity; and went to a well-known operator, with a note of introduction from me. He underwent the operation; and the stone was broken down at several sittings. Before returning to Feversham, he called upon me, and seemed feverish and ill; and I learnt subsequently, from Mr. Giraud, that he died, with typhoid symptoms, shortly after his return into the country. An abscess was formed in the neck of the bladder, at the base of the prostate gland."

*Case* 6. "A gentleman from the country, a patient of Mr. Elliott, of Stratford, was discovered to have stone in the bladder, and consulted me respecting it. Finding it to be small, I proposed to break it down; and employed Charrière's drill-instrument for the purpose. Meeting with several obstacles in the attempt, I abandoned it; and he afterwards was placed under the care of the same lithotritist, who broke up, as I was afterwards informed, a small phosphatic calculus. The patient left town with a very irritable bladder, and suffering much in voiding his urine. I subsequently heard, from Mr. Elliott, that he lived for twelve months, with increasing suffering.—On his death, the bladder was discovered to be diseased, and several stones were found in it."

*Case* 7. "Admiral C—— wrote to me from Exeter, to make some inquiry respecting the operation of lithotomy; as he was suffering from a calculus in the bladder. I replied to his inquiries, and was requested to make the necessary arrangements for cutting him. In the meantime, I was informed, by a friend, that he had placed himself under the care of the same lithotritist, and that the stone had been successfully broken down. Admiral C—— left town, but quickly experienced symptoms of a return of his disorder; and one or two fragments were, I believe, again detected, and crushed. He was thus relieved for a time; but experienced a second return of his pains, and died not long after his return into Devon. On the examination of his body by a surgeon at Exeter, six or seven calculi were found, formed, as I was told, on the remnants of the original calculus."

*Case* 8. "About nine years ago I operated on Mr. Saunders, of Tottenham, and removed a lithic-acid calculus. He recovered in a few days after the operation, which was performed with the assistance of Mr. Holt and Mr. Edward Cock. About three years ago he came to me again, with symptoms of stone; and, on sounding him, I discovered a small one, and told him that it was small enough to be broken down. He placed himself under the care of the same lithotritist, who crushed a small phosphatic calculus. He left town as soon as he was pronounced cured. Almost immediately after his return home, his pains in making water increased so much, that he said the pain of the stone was as nothing. He became feverish; and his bladder would not retain urine for an hour. I saw him, at the request of his surgeon, Mr. Moon, and thought that he might have some fragments remaining in his bladder. I passed a catheter, but could not detect any. His extreme irritability of bladder prevented any accurate examination by injecting it with warm water. He was averse to this latter measure, from the pain which he had always suffered when it had been formerly done. He died without any mitigation of his symptoms taking place."

*Case* 9. "Mr. S——, a patient of Dr. P——, was found to labour under retention of urine, caused by the presence of a small calculus in the bladder. He was lately placed under a lithotritist's care, for the purpose of operation. The stone was crushed, and the patient supposed to be cured. I subsequently met Mr. S——, at the house of Dr. P——, and heard him describe symptoms that indicated the presence of fragments in the bladder. I expressed this opinion to him; and told him to lose no time in applying to a lithotritist. Whether any further attempt was made to relieve him, I did not learn; but he was seized with bleedings from the bladder, which, joined to the irritation that he experienced, in a week or two destroyed him. On a post-mortem examination, several calculi were found in the bladder; and each was discovered, when broken, to be formed on a fragment of the crushed stone. A fungus had also shot forth from the cervix vesicæ, from which the bleeding had taken place."

The next three cases were considered by an eminent lithotritist, unfit for the operation of lithotrity, and were subjected to that of lithotomy with success. Such cases, of course, form an important element in any comparison of the two operations."

*Case* 10. "I was desired to visit Mr. N——, aged 70, with Mr. Burrows, surgeon in the City, in order to ascertain if he had a calculus. He had been supposed to labour under stone, but it had not been detected. On sounding him, a calculus was immediately struck by the instrument, and gave me the impression of being large. I explained to him the different operations that might be performed for his relief; and he gave the preference to lithotrity. The same operator met us at the patient's house; and sounded him with care, in order to ascertain the size of the stone, and the condition of the bladder. The former he discovered to be above the ordinary size; and the latter so irritable and contracted, that it would admit of a very small quantity of water only being injected. Under these circumstances, he was thought an unfavourable subject for lithotrity. I therefore removed the stone by the lateral operation, in the presence of the lithotritist and Mr. Burrows. The operation was difficult; and the extraction of the stone not easy, on account of its size, and the enlarged state of the prostate gland. He was convalescent at the end of the second week: and has remained, up to the present time (nearly five years), free from any symptoms of his former disorder."

*Case* 11. "I met Mr. Finch of Greenwich at the house of a gentleman nearly 80 years of age, who was much distressed by very urgent symptoms of urinary calculus. I was informed, that a lithotritist had sounded him, and, from his age, and the great irritability of his bladder, declined to operate.—His health being good, I saw no objection to lithotomy being performed, notwithstanding the enlargement of his prostate gland, and the irritable state of the mucous lining of his bladder. The operation was performed in the presence of Sir Richard Dobson, Mr. Finch, and Mr. Lawson: it was difficult, on account of the great enlargement of the body and third lobe of the gland; and tedious, from the number of stones, which were found to amount to twenty-seven. His convalescence was retarded by one or two small calculi passing by the wound; and by an abscess of the prostate gland, that burst into the rectum. The symptoms of irritation caused by this subsided; and when I heard of him, he was free from stone symptoms, and was in the enjoyment of good health."

*Case* 12. "Mr. C——, from Yorkshire, aged 60, came to town, for the purpose of placing himself under the same lithotritist's hands, for the operation of lithotrity. His bladder was so irritable, that water was with difficulty injected into it. After trying to allay the irritable state of the bladder for six weeks, the operator told him that his was not a case for lithotrity. I was requested to see him. I found him with an exquisitely sensitive bladder, affected with a bloody catarrh: his pulse 120; skin hot; and desire to void urine continual. The operation of lithotomy appeared to promise no success, especially as he had lost a great deal of flesh during his visit to town. I endeavoured to allay the irritation caused by the previous introduction of instruments, and succeeded to a certain extent. On sounding him, I discovered a large stone, and a large prostate gland. The operation was performed at the end of January, in the presence of Mr. Camplin and two young friends. It was difficult, on account of the stone being closely embraced by the bladder: it measured five inches and a half in its widest circumference. He was slow in recovering; the bladder still remaining irritable, and continuing to secrete a bloody mucus. The wound healed soundly, and his appetite and rest returned; and before he left town, the bladder had, in some degree, lost its extreme irritability, under the use of the uva ursi. I heard of him during the past Summer, and he was in the enjoyment of good health."

Such are the cases related by Mr. Key. So far as they go, they amount to this:—*that*, out of twelve cases of calculus in the bladder, nine were submitted to lithotrity, and three were handed over by the lithotritist to lithotomy; *that*, of the nine operated on by lithotrity, three only were cured, and six were totally unsuccessful; *that* of the unsuccessful cases, all proved fatal at no very remote period after the operation, which in several was performed on a small stone, and, *apparently* under favourable circumstances; *that*, in two of the unsuccessful cases, the fragments of the original calculus were found, after death, to have formed the nuclei of other calculi, although in each case, mark that, the patient was said and supposed to have been cured; and *that*, the three cases abandoned by lithotrity, and considered, therefore, as unfavourable, were all cured by lithotomy.

Mr. Key, after some remarks of little moment, observes that he wishes to shew that—

"As an exclusive operation for the removal of stone from the bladder, lithotrity cannot maintain that rank which it has been made to assume; and that if indiscriminately adopted, to the abandonment of lithotomy, it would be found inferior to the latter operation both in safety and success. The new operation should, on no account, be regarded in the light in which its exclusive advocates would place it—that is, as a substitute for the old; but as a valuable adjunct to it, and as furnishing the surgeon with an additional means of relieving a most alarming and painful disease. If this view be taken of it; it will, I think, be found to be one of the most useful inventions that modern surgery has added to her resources: for in many cases, if selected with judgment, it is not only equally effective with lithotomy, but more safe, and less painful in the performance."

Mr. Key goes on to state that the substantial benefits conferred by lithotrity, may be almost comprised in a few words, and in one sentiment. It has induced patients to apply with small stones, and it has furnished the means of detecting and of removing them when small. This is an opinion which has for some time been insisted on by the best-informed surgeons, and which is not new on the part of Mr. Key. As a proof of this we may cite some observations of our own published in October of last year.*

"When we sum up these cases forbidding, or, at the best, exceedingly unfavourable to, the operation of lithotrity, we shall probably find that they form a large proportion, if not the majority, of those which occur in hospitals. The lower class of persons, whose sensibilities are not usually acute, and who have to gain their livelihood by labour, seldom apply for medical advice until the complaint has existed for some time, and the calculus has either attained large dimensions, or the bladder or kidney is more or less diseased. We leave then the majority of cases for lithotomy, and we leave the *worst*. So far is lithotrity from being free from danger, that it abandons the bad cases to the knife. This is a truth that should be generally known. The direct advantages accruing from lithotrity have been greatly overrated, are, perhaps, overrated now, while, singularly enough, the indirect advantages are not sufficiently understood or prized.

Lithotrity has already done much for humanity, because in the first place it has led persons, with stone in the bladder, to apply earlier for medical advice, from the belief that the operation is not a severe one; in the second place, it has enabled us, by its improved modes of sounding, to detect small stones more readily than formerly; and, in the third place, it has given us the means of removing those stones, *while small*, with comparative facility and safety.

If lithotrity is inapplicable to large stones, it finds them out before they are large, and it removes them before they become so. This is the real value of the operation, the positive benefit it confers upon man. And no mean benefit. Who that had a small calculus in his bladder would be cut for it? What professional man would dream of suffering lithotomy in his own case? None, we are sure, aware of all the facts, and of the actual state of the question.

In the middle and the upper classes, where symptoms soon attract attention and means admit of the patient procuring good medical advice, small calculi are constantly discovered, and are now commonly removed by surgeons in this Metropolis. The bladder-forceps are introduced, after the bladder is injected with warm water, and the same instrument may sound the patient, detect the calculus, and either remove it whole, if it is small enough to be withdrawn through the urethra, or crush it by the screw or the hammer if it is not. How trivial, how simple, how safe is the operation, when weighed against lithotomy. When once the public grow well aware of the advantage of attending to the symptoms of stone, and of the benefit resulting from early medical attendance, we may anticipate that the operation of lithotomy, as well as lithotrity, under unfavourable circumstances, will be in a great measure superseded."

Mr. Key proceeds to institute a comparison between lithotomy and lithotrity. He adopts the following method. First, he points out the peculiar dangers of lithotomy—secondly, the circumstances that render lithotrity practicable—thirdly, the method of performing it—and, fourthly, the dangers attending it. It must be owned that this is not the most exact method of comparison, supposing a strict comparison to be admissible. But we will follow Mr. Key.

*Dangers of Lithotomy.*

We are not sure that we need dwell very long on these. They are tolerably notorious.

1. Mr. Key observes, which has repeatedly been done, that children bear lithotomy well. The records of all hospitals shew that. But we are not sure of the correctness of the observation that follows the statement of this fact.

"Though these young subjects are happily not liable to infiltration of urine into the cellular membrane, or to peritonitis, they are exposed to another source of danger, from sudden prostration, under which their powers will sometimes sink. This depression oc-

* Lithotrity and Lithotomy—Med. Chir. Rev. pp. 472—3. Oct. 1836.

curs in those children whose parents accustom them, even at their tender age, to the pernicious habit of dram-drinking; a custom partly arising from the erroneous idea, that gin, by increasing the urinary secretion, tends to allay the irritation produced by the stone. Such children, when brought into the hospital, being subjected to the lowering effects of a milder and restricted diet, and to the action of brisk purgative medicines, are rendered irritable and susceptible, and can ill bear an operation that exhausts their energy, and is attended with some loss of blood. A small dose of syrup of poppies, with a tea-spoonful of gin, or whatever liquor they may have been accustomed to, soon restores the tone of the nerves and the vigour of the circulating system."

There is some vagueness in the expression that children are not "liable" to "infiltration of urine into the cellular membrane, or to peritonitis." They are not *so* liable to these affections as adults are, but certainly they are occasionally attacked by them. We have seen several children, under eight years of age, die from suppuration in the cellular membrane of the pelvis, and from low cellular inflammation, extending more or less to the peritoneum. The proportion of children who die after lithotomy, compared with that of adults who die after the operation, is a small one—but of those children who do die, our own observation would lead us to say that the majority are cut off by cellular inflammation.

2. *Hæmorrhage.*

A. This is seldom, for obvious reasons, considerable in the child; but we quite agree with Mr. Key, that, in the adult, "it is not ingenuous for lithotomists to deny the occurrence, or the danger of hæmorrhage."

"There are several sources that furnish the hæmorrhage; but, in all cases, the bleeding comes most freely from the upper angle of the wound. The higher therefore the incision is carried, the greater, *cæteris paribus*, is the probability of bleeding. The pudic trunk itself is, I apprehend, rarely wounded. The artery of the bulb, and the superficial perineal branch, often bleed profusely, especially the former."

This is now generally admitted, and in the normal distribution of the vessels, it is the artery of the bulb rather than the pudic trunk, which bleeds, and which is expected to bleed.

B. But, occasionally, there are irregular arterial distributions, an instance of which occurred to the late Mr. Shaw, and is probably familiar to many of our readers.

C. But Mr. Key does not notice the most frequent source of *fatal* hæmorrhage. We allude to bleeding from the vesical and prostatic venous plexus in old persons. This it is, which is much more commonly mortal than arterial hæmorrhage, and there are few lithotomists who have not lost a patient by it.

D. But, perhaps the sort of hæmorrhage which, in practice, is productive of most mischief, is not that which is dangerous in its immediate consequences.

"The arteries are not large enough to afford a hæmorrhage sufficiently rapid to induce fatal syncope; but the draining sometimes continues for hours; either backward, into the bladder; or slowly, by the external wound; gradually exhausting the patient, and incapacitating him for the struggle which he may have to undergo with visceral disease or inflammation. It is this secondary effect that constitutes the chief danger of bleeding after lithotomy, and usually takes place in patients who have slight enlargement of the heart and a large arterial system. The pulse is found to be full and large, even when the patient appears exhausted by the loss of blood: the lungs become gorged, and, under the depressed state of the nervous energy, become embarrassed in their circulation. The heart, thus encumbered by the difficult pulmonary circulation, relieves itself by serous effusion into the pericardium, and death speedily ensues. If inflammation come on, either in the peritoneum or the vesical cellular tissue, the prostrated powers of the patient disable him from setting up a barrier to the mischief by a salutary adhesive process: in the latter case, the action assumes the suppurative form, and cellular abscess is the fatal consequence; and in the former, a slight extension of the peritonitis, producing opaque serous effusion, is sufficient to induce fatal prostration."

We were not aware of the peculiar tendency to dropsy of the *pericardium*, as a consequence of hæmorrhage after lithotomy, nor indeed of hæmorrhage at all. Dropsical effusions are a familiar effect of impeded transmission of blood through the lungs, but the particular tendency of such effusions to occur in the pericardium is a fact which is comparatively novel.

3. *Infiltration of Urine into the Cellular Membrane.*

A dangerous consequence of lithotomy, on which we need not dwell.

4. *Injury to the Neck of the Bladder and Prostate.*

Mr. Key's observations on this head are sensible, and we make no apology for extracting them in their original form.

"If we may judge from experience, the prostate gland would appear to sustain without inconvenience the effect of laceration: and this the experienced lithotomist bears in mind, in the act of extracting the stone. But contusion is most disastrous in its effects: it appears to cause sloughing, and diffuse inflammation of a destructive character in the neighbouring structures. In making the incision, and in extracting the stone, the object of the operator should be, to make a sufficient aperture to allow the stone to pass without bruising the gland; and not to carry the knife too far laterally, so as to divide the deep perineal fascia, and thus expose the cellular basin of the pelvis. The knife, if used incautiously, exposes the patient to this danger. If the prostate be large, and the stone of considerable size, a large aperture is required; and if the substance of the gland be indurated, the lips of the incision do not yield. To obviate these inconveniences, the surgeon carries the knife more freely through the prostate, and thus adds to the risk of opening the deep fascia that of hæmorrhage from the arteries of the gland. Under these circumstances, I always look to the assistance which the blunt gorget affords, as obviating both the difficulties, without inflicting an injury of a serious kind upon the gland. This instrument, in the hands of the late Mr. Martineau, has had its merits fully tried; and its success is sufficient answer to any objection that can be urged against it. His object, in this part of the operation, was three-fold—to make a small incision in the gland with the knife; to enlarge the incision with the blade of the blunt gorget; and to take time in drawing the stone from the bladder. In so conducting the operation, the gland is not bruised, but torn: the slow introduction of the blunt gorget separates the fibres of the gland, and enlarges the incision as far as the deep fascia, without dividing it; for the fascia yields, and stretches, as the gorget enters the bladder. Sudden violence, in the act of introducing the gorget, is nearly as much to be deprecated as the violent extraction of the stone. The kind of violence which the blunt gorget, properly employed, inflicts, is well borne by the gland, and symptoms of high inflammation are rare, after such an operation.

The extraction of the stone is attended with more hazard to the gland. A nervous or a violent operator, feeling some resistance to his efforts, redoubles them, until he finds the stone obeying the force that he employs. If the opening in the gland be made with a cutting instrument alone, and be small, the stone is brought with difficulty into the incision, as the lips of the wound do not open. Additional force is then employed; and the operator, in ignorance of what he is doing, drags the gland before the stone, separates it from its attachment to the deep fascia, and brings it nearly to the external aperture before his efforts succeed. The consequence of such an injudicious proceeding is, to bruise the gland, to cause it to slough, and to render infiltration of urine almost inevitable, by the laceration of the deep prostatic connexions. The danger is, in part, prevented by the use of a broad blunt gorget, which enables the broad end of a large stone to enter the incision; and renders it obedient to the gentle but continued force of the surgeon. In this step of the operation, time must be given for the parts that embrace the stone to dilate: the muscular structure at the neck of the bladder, the firm substance of the prostate gland, and the deep fascia, will each stretch, and yield to the sustained efforts of a firm but gentle hand. In cases of enlarged and hard prostate, I have occasionally opened the incision while passing the forceps through the prostate, by separating the blades in a vertical direction: this, however, should be done without violence.

The gland is sometimes injured in withdrawing the stone, by the forceps embracing a portion of it, and tearing it away from the body. This accident happens when the central portion of the gland, or, as it is termed, the third lobe, is enlarged, and presents a narrow ridge at the entrance of the bladder. The forceps, where the blades diverge, close on this projection, as they embrace the stone; and the operator is unaware of it, until a piece of the gland is brought away in the blades. It may be avoided, by passing the finger below the forceps, and disengaging the gland from the forceps after the stone is seized. In my operations, I always pass the left fore-finger along the staff, after the knife is withdrawn; and reconnoitre the size of the incision, the texture and form of the gland, and situation of the stone. If the finger feels a projecting portion of gland that rises between the blades of the forceps as they are opened laterally, care should be taken to prevent it being entangled, by depressing it as the stone is brought forward. As soon as the stone enters the incision, the danger is past. I do not, however, wish to magnify the extent of this danger; for, in truth, it is less than might be expected. The injured portion is brought away, and not left to slough; and the surface from which it is brought is a lacerated wound, without contusion. In one case, that of an elderly gentleman, it happened to myself in operating; but the patient, after suffering for a few days with irritative fever, ultimately did well: and I have observed, in the few cases that have occurred in the operations of other surgeons, that a slight degree only of fever, with fetid urine, has been the consequence, and in a few days has passed away. The neck of the bladder becomes slightly inflamed; but the incision prevents the ordinary severe effects of cystitis, by allowing the urine to drain away—an advantage that lithotrity does not possess.

The body of the prostate may receive injury from being grasped in the forceps, by the operator expanding the blades before they are fairly in the bladder. One blade slips beneath the lobe, while the other enters the bladder; and the operator, on closing them, thinking that they embrace the stone, uses considerable force in his attempt to extract it. The contusion and pressure which the gland undergoes are sufficient to induce inflammation, and, probably, some of its worst consequences."

5. *Peritonitis.*

On this we need say no more than that, frequent as it is, in some degree or other, both its frequency and its degree are probably exaggerated. The most obvious cause, and in all likelihood, the true one, is extension of inflammatory action by the cellular tissue to the peritoneum.

If proper care is used, fragments need seldom, if ever, be left in the bladder after the operation of lithotomy. But if any *are* left, they usually come away by the fourth day, when the tumefaction occasioned by the incision has subsided.

Coagula often collect in the bladder, through the oozing from the prostatic vessels; but occasion sufficient irritation to induce the bladder to expel them. The patient may be rendered very uneasy after the operation, by a sense of distention in the region of the bladder, and of bearing down in the perineum; which continues until the repeated efforts of the bladder expel some small masses of coagula: or, if the distress be great soon after the patient is put to bed, and continues toward the evening, it is often found to arise from coagula obstructing the flow of urine; and the gentle introduction of the finger along the wound is followed by a copious discharge of urine mixed with coagula.

7. *Circumscribed Abscess in the Pelvis.*

Mr. Key makes no mention of this consequence of lithotomy. We have seen two or three patients die of it.

8. *Wound of the Rectum.*

This occasionally happens, but is seldom of much consequence, the wound, in most instances, gradually healing.

9. *Fistula in Perinæo.*

Mr. Key has not seen this after the extraction of a vesical calculus. But it sometimes follows the removal of prostatic calculi.

"In a gentleman," says Mr. Key, "verging upon eighty years of age, whom I cut about three years since, an abscess formed in the prostate gland, and burst into the rectum. Benefit, rather than inconvenience, followed this; for the prostate, being very much enlarged, had obstructed the free escape of urine from the bladder, and partial retention often occurred: the aperture into the rectum remedied the difficulty, by allowing the bladder to empty itself more completely; and was otherwise of little inconvenience to him."

10. *Impotence.*

Mr. Key has known this happen in one instance. Other cases of the sort have been reported, and it is singular that it is not a more frequent result of the operation.

11. *Incontinence of Urine.*

This is rare in the adult. In the young subject, partial incontinence will sometimes occur, if the patient is allowed to leave his bed too soon after the operation, before the neck of the bladder is firmly healed, and the sphincter has recovered its tone. Instances, therefore, are met with, of young boys, who, if they retain their water inconveniently long, find it dribble away as they move about. In bed, the urine is perfectly retained. When they arrive at the age of puberty, the power of retaining it becomes increased.

12. *Vesical Phlebitis and the Secondary Inflammations.*

Mr. Key takes no notice of these affections. They may occur conjointly, or separately. Many fatal cases have been at different times reported, and we have ourselves seen two.

Such is the list, a formidable one on paper, no slight one in practice, of the dangers that wait upon lithotomy.—Though that operation is much more successful in the hands of English surgeons, than the contemplation of the risks which patients run might induce us to suppose, yet candour must admit that a safer substitute for this operation would be a boon to humanity.

LITHOTRITY.

Lithotrity was proposed as such a substitute, its claims were specious, its advocates were confident, perhaps, not quite ingenuous, and can we wonder that men caught at what seemed a blessing, unalloyed by anything of evil? As usual, there were two parties in the profession. One, sanguine and enthusiastic, was charmed with the novelty, and deluded by the bold promise of lithotrity. With that party all was couleur de rose, and whoever did not think exactly as it thought, whoever did not instantly abandon the old method and adopt the new, was either a bigot or a fool. This party in science is not a small one, because it comprises the worst-informed and the least experienced. Its humour and its violence have frequently reminded us of the picture that has been drawn by a master's hand, of a similar faction in politics:—

A headstrong, moody, murmuring race,
As ever tried the extent, and stretch of grace;
God's pampered people, whom, debauched with ease,
No king can govern, nor no God can please.

Another party, which has assumed the office of drag on the scientific wheel, and has enjoyed the honour of resisting to the uttermost and to the last the improvements in modern medicine, denounced lithotrity as absurd and mischievous, and proved to demonstration that that could not possibly be done, which somehow or other, was done daily.* But this party had been broken by former defeats, and its voice was soon lost amidst the shouts of its opponents.

More moderate men, the more moderate because the more experienced, for experience leads to moderation in medicine, were struck with the force of much that the lithotritists advanced, while they could not avoid seeing many strong objections to their methods. The pretension to the absence of all danger from the operation was contrary to reason, and was soon contradicted by facts.† The subsequent modifications of the process have established the soundness of many of the exceptions that were taken by well informed surgeons to lithotrity, and the progress of time, so favourable in every case to truth, has brought to light facts which may serve as a basis for a just appreciation of the new operation.

But this is too wide a question for us at present. Mr. Key cannot fairly be said to have met it, for the remainder of his observations refer rather to the mode of performing the operation, and consist of miscellaneous hints. We shall endeavour to pick out what seems to us to be possessed of novelty, or to be of value on other accounts.

1. *Sounding the Bladder.*

Mr. Key's remarks on this head are judicious. He rightly observes that lithotrity has done much to improve our method of sounding, and that Heurteloup's bed is a material assistance. A small quantity of water, generally about three ounces, should be injected into the bladder, a much larger or a much smaller quantity being alike disadvantageous. In order to make the examination more satisfactory, the stone should be dislodged from behind the prostate, by suddenly inclining the patient backward, and altering the axis of the bladder: this is effected by the couch or chair.

Though touch will usually decide the presence of a calculus, the surgeon should not be satisfied until he has heard it.

"I have found sometimes, when the friction of the sound over the foreign substance has been equivocal, that the injection of oil into the bladder has removed the deceptive sensation which the contact of metal with a fungoid mass surrounded by water will produce. An instance occurred not long since, of a bleeding fungus being mistaken for a calculus. The patient was sounded more than once, by an experienced surgeon, and pronounced to have stone: he was placed under the care of a lithotritist, who fixed a day for the operation. The patient, however, became so ill, before the day arrived, that it was necessary to defer the operation. On his death, which took place in a few days afterwards, no stone was discovered in the bladder; but a bleeding fungus, of considerable size, was found attached to the mucous membrane.—The occurrence of a cyst enveloping the stone in the bladder is often held out as likely to mislead the surgeon, and to prevent that satisfactory evidence being obtained that a careful operator would wish to have before proceeding to the operation. The occasional presence of a cyst around a calculus cannot be denied. In a case on which Mr. W. Wickham operated, a something was removed with the stone that resembled a cyst formed by adhesive matter; it was so thin, as not to prevent a distinct sensation being communicated by the sound. Such cases are very rare. The stone may also be lodged in a sac formed by a protrusion of the mucous coat between the muscular fibres of the

* Everybody knows how ingeniously it was contended, that a straight instrument could not work in the bladder, at the very time that many people were working it.

† We believe we took a fair estimate of lithotrity at an early period; and we were the first, in this country, to publish a series of fatal cases.

bladder. Such a variation in the position of the stone is seen sometimes. We have a specimen of it in the Museum; but, like the former, it is a very unusual variety in the situation of a vesical calculus. I have never met with such accidents, either in hospital or private practice; nor have I witnessed them in that of my colleagues."

The steel sound and the silver catheter are the two instruments used for sounding. Each has its advantages.

The best form of catheter is Heurteloup's. The sensation conveyed by the catheter is less distinct than that transmitted by the sound. That is, of course, a disadvantage.

"Another disadvantage of the catheter is, the alteration that it produces in the course of the canal, by raising the prostate gland. This will be understood by contrasting the straight tube of the catheter with the curve of the sound. When the former is in the bladder, the part of it that lies in the prostatic portion of the urethra is a straight line; and when the hand is depressed, the prostatic portion is necessarily raised, and the *cul de sac* of the bladder anterior to the ureters is deepened. A small stone, or fragments, may then escape the touch of the catheter, unless they can be thrown backward into the base of the bladder. The common steel sound is free from this disadvantage: when the beak is in the bladder, its curved portion is in the prostate gland, and causes no distortion in that part of the canal; so that, as it enters the bladder, a small stone may be struck, over which the silver cathether would pass without touching it. If the bladder contains about an ounce of urine, which is sufficient to allow the sound to enter freely, the stone, however small, rarely eludes it. In the examination of persons suspected to be afflicted with calculus, I usually first employ the catheter, after injecting two or three ounces of warm water, and reclining the chair: and if I do not strike a stone, or if I meet with a something, of the nature of which the silver tube fails accurately to inform me, I then allow some of the water to escape, and introduce a common steel instrument."

2. *Subsequent Steps of the Operation.*

For these we may refer to Mr. Key's paper, or to the works upon lithotrity. M. Key has modified in a slight degree Baron Heurteloup's "lit rectangle." He has had it so constructed as readily to be taken to pieces, for the convenience of travelling. It is made of beech, with a bedding of common ticking, which can be rendered more easy to the patient by a pillow behind his back: the pelvis sinks sufficiently to bring the urethra nearly on a level with the vice; which thus requires not to be so much raised, and is thereby rendered steadier. The fore legs are about an inch and a half longer than the hind ones, in order to give a more convenient direction to the percussor when fixed in the vice, and also to give a greater inclination backward to the pelvis.

Mr. Key has also modified the lithotritic instrument. An objection to lithotrity is the necessity for frequent sittings, in consequence of the fragments escaping from the instrument, and requiring to be severally broken up. To prevent the escape of those fragments, when too large to pass through the urethra, has been Mr. Key's object.

"Accident led me to contemplate the possibility of adapting a net-work, for the purpose not only of catching the fragments, but also of throwing them between the teeth of the percussor, in the act of withdrawing the male blade. Mr. Laundy carried into effect the idea that I had suggested to him, and proved that an instrument might be so constructed as to effect both these desirable ends.

The percussor that seemed to be the best for my purpose was that made by Weiss: the great power that it possessed over a calculus, and its property of clearing itself, were two advantages that no other lithotrite, that I had seen, possessed; and, at my request, he constructed a lithotrite with an addition of two branches, projecting by means of wires concealed in a groove at the upper part of the female blade. *Fig.* 1. represents Weiss's lithotrite, with the branches added to it. They move by means of a handle attached on each side of the instrument. When the instrument is closed, the handles are fixed by a screw and eye to the upper part of the male blade: the branches are concealed when the blades are closed; but are seen lying close to the sides of the male blade when the latter is withdrawn. The branches are pierced with small holes, for the purpose of fixing the net, as is also the posterior part of the male blade itself, as is shewn in *Fig* 2: one end of the net being fixed to the blade, and the other to the branch, when the latter is projected, it expands the net on each side of the calculus. The net occupies so small a space, that, when gathered up, it lies close to the side of the blade, which is slightly excavated to receive it, and does not interfere with the blade closing perfectly. See *Fig*. 3.

When the instrument is closed, ready to be introduced into the urethra, the blades appear in no way to differ from those of the ordinary lithotrite, except at the back of the female blade; in which I have had introduced small bars, so as to intercept large fragments, and prevent them from escaping before they are sufficiently triturated: these are seen at *Fig*. 4."

This description cannot be thoroughly understood without reference to the plate which accompanies it. But those who feel anxious on the subject will probably procure the instrument itself. It essentially consists of two slender lateral branches, supporting a net.—Whether the instrument will answer must be left to experience to decide. But some plausible objections may be brought against it. Mr. Key has employed it in four cases; in two or three, he has been obliged to lay it aside and use the common lithotrite.

Leaving the mode of performing the operation, we will touch on two or three circumstances which affect it, and which increase or diminish the patient's chances of recovery.

A. *Size of the Calculus.*

Mr. Key observes, that the size of the calculus forms of itself no objection to lithotrity. Now this, as an unconditional proposition, is opposed both by theory and practice. Mr. Key, himself, is obliged to modify it, for he observes that "he knows of no limits to the size of a calculus removable by lithotrity, but the power of the lithotrite." But that itself forms the limitation, and hedges in the proposition, which would otherwise be so broad an one. In surgery, it is not absolute possibility which determines the performance of operations, but a calculation of probabilities. A large stone may be broken by the lithotrite many times without an accident, but then the lithotrite itself may give way, and no prudent surgeon would incur that hazard. It is no answer, to urge that Mr. Costello has broken up a calculus weighing several ounces. "One swallow doesn't make a summer," and a general proposition cannot be established by a particular case or two. It is well known that lithotritists, and those the most able and experienced, will *not* generally meddle with large stones, a pretty sure proof that to do so would be dangerous.

Mr. Key justly observes, too, that there are circumstances, more or less attendant on large calculi, which should be carefully weighed by the surgeon.

"As the stone cannot be entirely crushed at one sitting, a patient with an irritable or an unsound bladder becomes involved in most serious danger by the operation being hastily adopted. A large stone broken up into many irregular fragments, all crowded by the contractions of the bladder against the irritable and inflamed cervix, causes excessive efforts to void the urine, and even inflammation of the mucous surface. Under such circumstances, the repetition of the operation becomes impossible, or highly dangerous; and the patient has to struggle through the stages of inflammation, with a bladder irritated by the lesser fragments. But if the bladder be free from disease, and not very irritable, it will bear the number of sittings required to break up a large stone, without much suffering to the patient, and with very little danger."

When we take into consideration, *that*, when the stone is large, the bladder is frequently not healthy—*that*, the operation is rendered proportionately more troublesome and more tedious—*that,* repeated operations are required—*that,* such operations and the presence of fragments are very likely to produce inflammation of the bladder, even if it did not previously exist—and *that,* all these circumstances make the operation both a painful and a dangerous one,—we may readily comprehend the general reluctance on the part of lithotritists to engage with large stones, and the general preference, which, in such cases, is, and should be given to lithotomy.

B. *Lithotrity more or less inapplicable to early and advanced life.*

This is a trite axiom. It is comparatively inapplicable to youth, on account of the small size of the urethra—to old age, on account of the usual presence of enlargement of the prostate. But if in youth, there should be a very capacious urethra, or if, in old age, the prostate be small, the principal objections to lithotrity in those cases would be done away with.

C. *State of the Bladder to be considered.*

"Three conditions of this organ are necessary; and these must be ascertained, by preliminary observations and trials, before the operation is determined on:—1st, It must be capable of holding a sufficient quantity of water to facilitate the working of the percussor. 2dly, It must be free from that extreme irritability that often attends the latter stages of calculous disorders; and, 3dly, Not prone to inflammation, from slight excitement. In healthy persons, the bladder, even under the irritation of a stone, will allow several ounces of water to be injected into its cavity, without sustaining more than a slight inclination to eject it. Its retentive powers are not impaired in the early stages of the disorder: patients will go for many hours without any desire to empty the bladder, the only early symptom being a smarting, when the bladder contracts on the calculus. It is therefore rare to meet with any difficulty in injecting water sufficient for the purpose of giving space for the operation, among those who apply for advice soon after the symptoms have begun to declare themselves.

Even when, from the long-continued presence of the stone, the bladder becomes morbidly affected, and able to contain but three or four ounces of urine without an irresistible desire to expel it, much may be done, by treatment, to assuage the irritation of the mucous membrane, and tranquillize the muscular excitability. When the stone has been long resident in the bladder, and has produced a change in the mucous membrane and a copious discharge of phosphatic mucus, signs of extreme irritability come on, and almost seem to forbid any expectation of lithotrity being practicable. The desire to void urine is renewed every two hours, or oftener: the urine not only deposits a large quantity of dark-coloured mucus, but is cloudy, and loaded with small flakes of adhesive matter, the result of inflammation of the mucous lining: the pain in expelling the last few drops of mucus is intense. Such continued suffering at length affects the general health; and would seem, I say, to forbid the operation altogether. Frequently, however, will these formidable symptoms yield to a system of diet and medicine, and the patient by degrees be unexpectedly brought into a condition to bear the operation."

Mr. Key remarks, what every surgeon has observed, that those who have irritable bladders, usually have some disturbance after moderate distention with water and examination by the catheter. This disturbance is commonly evinced or accompanied by a rigor. Unless there is persistent pyrexia, this is not to be considered as alarming. Every body knows, that the same thing frequently occurs after the introduction of bougies.

D. *Morbid State of the Cervix Vesicæ.*

"One principal source of irritability of the bladder is a morbid condition of the cervix or of the prostate gland.—The structure about the neck of the bladder, above all others deserves the especial attention of the lithotritist; as it is here that he will meet with the most difficulties, and will also find the chief source of danger. The extreme susceptibility of this part of the bladder is not unfrequently evinced in severe rigor, and inflammation following the introduction of a sound in patients who complain of dysuria connected with an enlarged prostate gland. These persons, often highly disposed to inflammation, have a severe attack, brought on by the casual introduction of an instrument for the purpose of ascertaining the cause of their ailments. When the morbid condition of the gland is combined with calculus, the risk of inflammation, and the danger of its consequences, become greatly increased; and the hasty performance of lithotrity in persons not prepared for the operation has been known to induce a fatal cystitis. Too much caution cannot be used in ascertaining the exact condition of the neck of the bladder, by occasional examination with the sound; not only with the view of learning the physical state of the gland, but also of measuring the degree of susceptibility. The irritability of the cervix, instead of being increased by the catheter, is often considerably diminished by the careful and occasional introduction of a large instrument, more especially if assisted by regulated diet and alkaline saline medicines; whereas, if the operation be performed without such preparation, on a person enjoying high health, the combined efforts of the sudden distention of the cervix and prostate gland, the reduction of a hard stone into angular masses, that are propelled, at each time of voiding urine, into the prostatic part of the canal, and the natural susceptibility of the bladder, bring on a degree of inflammation that is with difficulty arrested."

E. *Calculi in the Kidney.*

"The presence of calculi in the kidney," says Mr. Key, "cannot always be ascertained before the operation is performed; nor does this seem to be important." We subscribe to the truth of the first half of the paragraph, but not to that of the second. We believe it is a matter of considerable importance, whether there be or be not calculi in the kidney. The affections of the kidney, both after lithotrity and lithotomy, are much to be dreaded, and are too often fatal. To the surgeon, the condition of the kidney, in every point of view, must be an object of anxious concern.

This article has run out to a considerable length. In the original, it occupies 56 pages. It is, in a great degree, addressed to students, and much of it must be familiar to surgeons of information or experience. We have omitted many details, and selected those points which were of highest interest for notice here.

The paper cannot be considered a comparison between lithotrity and lithotomy. Such a comparison is difficult in some respects, impossible in others, for every day's experience is shewing forcibly that the two operations are not adapted to the same cases. Nor do we think that the paper presents a complete account of the bad consequences of lithotrity, nor of the indications for either operation. But there are many practical and useful hints in it, and the subject is one which, at present, engrosses, and deserves to engross, the attention of the surgical world, and which neither is, nor for some time to come, will be exhausted.

### II. Two Cases of Poisoning by Arsenious Acid. By Mr. Alfred S. Taylor.

Appended to these cases, which are, themselves interesting, are some good remarks on the solubility of arsenious acid in water and in other menstrua. We have not space for the latter observations, and must confine ourselves to a notice of the cases, and to their chief bearings on forensic medicine.

*Case* 1. A girl, aged 25, took arsenic, probably about forty grains, about four o'clock in the afternoon of the 17th of May, 1836. She became very ill in about an hour afterwards, and had then pain in the stomach. About seven hours after taking the poison, when admitted, there was great thirst, but the pain in the stomach was not great, nor increased by pressure. The thirst continued unabated till her death, which took place at a quarter past seven in the morning, a little more than fifteen hours after having taken the poison. She was sensible until just before her death.

*Dissection.* The bronchial lining membrane was rather congested and dark.

On opening the abdomen, and exposing the cavity of the stomach, this organ was found to contain a dirty yellowish-coloured liquid, somewhat tenacious, and holding, diffused through it, opaque grains of a white matter, evidently arsenic. Particles of the same substance, enveloped in mucus, were found scattered over the surface of the mucous membrane, in different parts. The appearances presented by the stomach, when seen and examined shortly after its removal, were as follows.* The mucous membrane was very rugose, especially at the larger extremity and inferior portion. The edges of the rugæ were, for the most part, vascular; whilst, in the intervening depressions, there were here and there dark coloured patches of blood, extravasated beneath the mucous membrane. There was great vascularity of the membrane in the upper part of the organ; and there were three distinct lines, of a vermilion redness (the colour having become a little more intense by exposure), running parallel to each other, for nearly the whole of the interspace between the pyloric and the cardiac orifices. These lines terminated in a well-defined margin of redness, situated at the junction of the cardia with the œsophagus.

The most striking morbid changes, however, in the stomach existed near the larger curvature, towards the pyloric extremity. Here there was a large prominent oval patch of thickened membrane, about three inches in length, and two inches in breadth. This patch was, in the first instance, covered with a dense layer of opaque mucus, with difficulty separable, containing small granules of arsenic diffused in a white pasty mass. When the surface was washed, it was seen to be of a yellowish colour in the centre; and it was surrounded by a dark margin, as of extravasated blood. At this part, the coats of the stomach were at least three-quarters of an inch in thickness. There was no trace of ulceration or corrosion in any part of the mucous membrane. The peritoneal coat was slightly injected.

In the report of the examination, it is stated that the small intestines contained a great quantity of viscid mucus, tinged with bile. The duodenum was but slightly vascular; but the jejunum was in a high state of inflammation, in circumscribed patches, these portions of the mucous membrane being covered with an easily separable layer of mucus. The lining membrane of the last twelve inches of the ileum, as well as that of the cæcum, was slightly inflamed. The rest of the intestinal canal, with the other organs of the abdomen, presented no abnormal appearances. From the condition of the uterus and its appendages, there was reason to suspect that impregnation had recently taken place.

Mr. Taylor points out as remarkable features in the case:—1, the general absence of pain; 2, the extreme thirst, a symptom on which he insists, as characteristic and valuable; 3, the presence of extreme thickening, and the absence of ulceration.

*Case* 2. A female, aged 22, was admitted at 9 p.m., Sept. 3, 1836, having taken, so it was reported, an ounce of arsenious acid, one hour previously. First, she was made to vomit freely, by various means, then she was seized with violent diarrhœa, then had extreme prostration, then grew comatose and was affected with cramps in the legs, and died seventeen hours after taking the poison.

*Dissection* 26 *hours after death.* The mucous membrane of the stomach was highly inflamed throughout, and was in

* A wax model of the stomach, representing very perfectly the morbid changes observed in it, has been made by Mr. Town. It is placed in the Museum, and numbered 2772[c].

many parts *ulcerated.* The rugæ of the membrane were not very numerous, but their summits were especially the seat of inflammation and ulceration. No *arsenic was found in the stomach,* nor were there any solid matters present.

The mucous membrane of the duodenum was much inflamed, and there were also considerable patches of inflammation and ulceration on that of the ileum. The mucous membrane of the colon was slightly injected, as well as that of the rectum; but there was no trace of poison to be discovered in the last-mentioned portion of the intestinal canal. The œsophagus was likewise inflamed, and the lining membrane of the larynx and trachea was highly injected. The uterus exhibited evidence of existing menstruation.

In this case there *were* ulcerations, there was *not* thirst, there was *no* trace of poison discoverable in the stomach or intestines, but arsenic *was* detected in the matters vomited.

For the rest of Mr. Taylor's paper we must refer our readers to the original reports. In our next number we may glance at the prominent features of it.

### III. On the Safety-valve of the Heart.

Nor will our limits permit us to do more than mention an Essay, of 77 pages, by Mr. King, on the safety-valve function in the right ventricle of the human heart. In our next number we shall present an abstract of its leading facts, but anatomy and physiology have had their full share in the present.

### IV. Reparation of Fractured Bones.

Eighteen pages and several plates are devoted by Mr. Bransby Cooper to this interesting and important subject. This memoir has been reviewed in another part of this number.*

* Review Department. Art. Pathological Anatomy.

### V. Cases and Observations illustrative of the Diagnosis of Tumors at the Basis of the Brain, or of other Lesions of the Brain and Cord.

All are disposed to attach value to whatever pathological remarks proceed from our able friend, Dr. Bright. We cannot then defer to a more remote opportunity the examination of this paper.

### A. *Tumors at the Basis Cranii.*

Dr. Bright observes that in disease, as in other things, there must be a fixed relation between causes and effects.— This may not unreasonably be granted, but the disturbing circumstances are sometimes so numerous or so powerful, that it is difficult, if not impossible, accurately to appreciate them. Hence the mistakes in diagnosis, hence the caution of experienced practitioners, caution founded on the jockey principle, "once singed, twice shy!" The difficulties and the embarrassments attendant upon diagnosis are most keenly felt, for obvious reasons, when the nervous system is concerned. Should those difficulties be diminished, a material boon would be conferred on medicine, and no small thanks will be due to Dr. Bright if he assists in their reduction.

In each of the two succeeding cases, a tumor was found, just beneath the tentorium, in contact with, or actually attached to, the petrous portion of the temporal bone, and pressing aside the pons varolii.

*Case* 1. In Nov. 1831, Dr. Bright states, and we must use, for we cannot usefully abridge them, his own words, that he was sent for to Woolwich, to see an officer, a tall athletic man, aged 48, who, after being many years in active foreign service, had returned home in 1817. He married in 1825, and had a family. In 1826, he suffered severely from sciatica of the right hip, which he had injured some years before, by a fall from his horse. This was cured by carbonate of iron; and he afterwards enjoyed an excellent state of health, till the Autumn of 1829, when, being again engaged in foreign service, he began to experience an attack of periodic pain over the left eye, exactly in the super-orbitar notch. This pain used to return about dinner-time, daily; and was always put a stop to before he had half finished his meal. At this time he met with a very severe accident; and was taken up, stunned, and senseless. He was bled; and, after some hours, recovered; but his recollection was, for a day or two, so defective, that he frequently asked what service they were engaged in, and what they were doing; and it was with great difficulty these points could be explained to him. However, in a short time he was tolerably restored; though he never regained the state of health he had enjoyed before his fall, frequently complaining of pains in his head, and of some weakness in his right leg.

About Christmas, he had a severe attack of bilious vomiting; after which, the pains in his head, and over the right eye, were worse, and more frequent; and he experienced, occasionally, a temporary loss of sight, coming over him like a cloud, and lasting for some minutes; then passing off, and not being followed by any remarkable increase of the headache. After some weeks of suffering, the intermittent pain over the left eye was completely removed in a single day, by taking three doses of sulphate of quinine in rapid succession, two or three hours before the expected diurnal attack; and it never afterwards returned. In June 1830 he had another attack of bilious vomiting, of great severity; which, however, passed off so quickly, that the following day he was able to resume all his duties; but shortly afterwards he discovered, one morning, that the sight of the left eye was completely gone. The sight of the right eye also became imperfect; the weakness of the right leg increased; and the left leg also began to lose power. In 1831, when he returned home, the sight of one eye was entirely lost; and with the other, it was only by great effort, and by changing the field of vision often, that he could discover the features of any one with whom he conversed. The hearing of the right ear was tolerably perfect; but he had, for many years, lost the hearing of the left, from the shock of a gun firing. He likewise complained much of pain darting through his head; which was relieved by cupping, blistering, and tartar-emetic ointment.

Dr. B. first saw him on the 8th Nov. 1831. He was then sitting in his chair, perfectly unconscious of surrounding objects, nor was he aware of Dr. Bright's presence. When, after much trouble, by writing words on his hand, and by calling in his ear, and by other means, he was led to comprehend, he answered distinctly, and without hesitation, but in the high-raised and ill-modulated voice which is usually observed in deaf people. His intellect seemed unimpaired. He was able to stand; but, partly from the weakness of his lower extremities, and still more from the timidity arising from his blindness, he could not move without support; and when he attempted to walk, it was with a short, feeble, tottering step. He had no incontinence of urine, although that had occasionally appeared some weeks before: he had never passed his fæces unconsciously, but once or twice there had scarcely been time to prevent an accident of that kind. His sleep was tranquil, and not too heavy; nor did he appear more drowsy than might be expected in a person deprived of sight and hearing. His appetite was good, and had sometimes been excessive.—There was some inequality in his power of hearing. At times he could catch sounds, but never distinctly, as to understand their meanings. About a week before Dr. B.'s visit, he had had a fit, with temporary insensibility and suffused countenance, but without convulsion.

In the course of November he partially recovered his hearing, being able once or twice to distinguish the drum or the bugle sounding, nay, even certain words. If made to hear what was said he comprehended it, and answered rightly. Dr. B. again saw him on the 29th. He had sometimes spoken of a very peculiar sensation in his head, attended with a sound as if grease had been thrown into the fire, making a whizzing noise and then dying away, whilst at the same time a flash of light passed over his eyes. His sight had also been occasionally improved. He was strong on his legs. The pulse varied from 70 to 75. Light bitters with the volatile alkali, blisters, and a seton were the means employed.

After this the symptoms gradually increased, and the mental faculties grew weaker. Dr. B. saw him again on the 14th of November, 1832. He was then in bed, helpless, and much emaciated. His wife was feeding him with meat, finely minced, and mixed with potatoes: she was obliged to rouse him frequently, to make him take his food; and then he continued to open and close his teeth gently, till he fell asleep, while the meal still remained partly in his mouth. Taste appeared to be lost. He was quite unconscious, and usually in a kind of slumber, but, on some days, was more drowsy than on others. If conscious of the calls of nature, he gave no intimation that he was so. The pupils seemed quite motionless. He occasionally expressed severe headache, and a pain over the right eye.

He died on the 27th of December. He had sunk progressively, and sloughs formed on the sacrum before death.

*Dissection.* The scalp rather more bloodless than usual. The scull was hard and solid, and somewhat uneven in its thickness: on each side of the sagittal suture internally, but particularly on the left, it had small, deep, irregular cavities, which had been filled with corresponding unusually-enlarged glandulæ Pacchioni, which seemed to have almost perforated the scull in some parts, so that only the external table remained. The dura mater was not very vascular; but the projection of the glandular bodies, on each side of the longitudinal sinus, was remarkable; so that, at first, they suggested the idea of small cerebriform fungous tumors. A small bony plate also, about half an inch in length, lay along the angle of the falx. The longitudinal sinus was quite natural. The dura mater adhered very firmly to the arachnoid, at those parts where the glands were so large; and when it was removed, the arachnoid in the immediate neighbourhood was white and opaque. The arachnoid was not vascular, nor unnaturally adherent to the brain. There was no serum effused beneath it. The depth between the two hemispheres of the cerebrum was small, owing to a considerable elevation of the corpus callosum. The general substance of the brain was natural, but rather deficient in bloody points.

The roof of the ventricles was raised high by clear fluid, of which about four ounces were collected, both the posterior and the anterior portions of the ventricles being distended; but the accumulation appeared greatest in the anterior. A few large vessels, ramified on the internal surfaces of the ventricles, the corpora striata, and the optic thalami, seemed flattened; and the septum lucidum was much thicker and firmer than natural. The choroid plexus, on each side, was exsanguine, and contained several vesicles, from the size of a pin's head to that of a pea. The velum interpositum was also exsanguine.

In attempting to remove the brain from the basis of the scull, it was found that the anterior portion of the cerebellum, on the left side, degenerated into a tumor; and adhered so firmly, that it could not be detached without a scalpel, or employing considerable force, from the petrous portion of the temporal bone. The structure of this tumor was chiefly hard and unyielding, but in some parts softer; and the nervus trigeminus, or fifth nerve, was seen passing over it, flattened and broad: nor did the tumor simply adhere, but the bone had become carious, and pervaded by it, so that a softened cavity occupied a large portion of the petrous ridge, extending towards the sella turcica.

*Case* 2. N. Wells, aged 43, admitted into Guy's Hospital, October 22d, 1834.

In 1817, he received a wound from the bursting of a gun, by which his cheek bone was much injured, and which was followed by defective hearing in the left ear. No other ill consequences followed until 18 months ago, when an offensive discharge took place from the left ear, which, after continuing for eight months, suddenly stopped about Christmas last. This was quickly followed by a pain across the forehead, and a sense of weight towards the back of the head. About four months ago, the vision of the left eye became imperfect; and in another month, the right eye was likewise affected.

At the time of his admission, the vision of both eyes had, for the last month, been so defective, that he was unable to find his way in the street. During the last six weeks, he had been attacked, three or four times almost every day, with a convulsive agitation, chiefly affecting the left side; and he stated that his memory had become very imperfect respecting recent circumstances, but was more retentive of those of earlier date. His pulse was variable in strength and frequency, generally about 96.

He remained under Dr. Bright's care for nearly three months. The treatment consisted in calomel, leeches, counter-irritation, &c.; but it was of no service. The chief variations which took place, during the time he remained, were the occasional recurrence of very severe headaches. He once or twice fell to the ground, in fits of giddiness; and was often unable to move, from what he called the heaviness of his head. He had occasional difficulty in passing his urine; and his bowels were costive. On the 13th of January, he left the hospital, being at that time totally blind; and never afterwards could distinguish light from darkness. He was quite deaf in the left ear, and the hearing of the right was at times much affected. He had then the perfect use of his limbs; and was in the habit of being led out for walks to a considerable distance. In this state he continued until the Summer of 1835, when he was suddenly attacked one day while walking, and fell insensible. He was attended by Mr. Griffith, a very intelligent surgeon, of Pimlico, who reports that after this :—

"He was hemiplegic on the right side, and the mouth was drawn to the left. On the side affected, there was total loss of sensation. He recovered, in some degree, sensation, and the power of motion in the arm and leg; but the effort to use either was always accompanied with violent shaking. He could not stand without support. He at all times had the power of retaining and voiding his fæces and urine: the bowels were very torpid, requiring the daily use of full doses of aperient medicine to procure relief. He was subject to convulsive attacks; which came on sometimes three times, sometimes only once a day, and sometimes with an intermission of a day or two. His mind was at times childish, but his reason was not gone: his sense of taste was entirely destroyed: his hearing varied, being sometimes very good, at others imperfect. Though I had not seen him for many months, he immediately recognised me by my voice. This was about two months before his death: he then articulated with much difficulty: he uttered his words suddenly, and after a prolonged effort.—His tongue was drawn forcibly to the roof of his mouth; and in the effort to speak, there was apparently great difficulty to depress the lower jaw: he frequently, however, gaped, and yawned to the full extent. It appeared that the muscles of the right side of the jaw had in a degree regained their power; for the distortion, when I saw him, was in no great degree: sensation, however, was entirely lost on this side of the face. He was a man of irritable temper, and passionate; but I did not observe that he was lately more so than usual. For a considerable time before his death, his arms were so paralyzed, that he could not feed himself; and for the last ten weeks, he could not leave his bed on account of the paralyzed state of his legs."

He died on the 24th of October, 1836, and was examined by Mr. Griffith, in the presence of Mr. Dewsnap, Mr. Bowling, and Dr. Bright.

*Dissection.* On raising the calvaria, the dura mater appeared tense, but not remarkably vascular. Two or three large glandulæ Pacchioni stood out on its surface, by the side of the longitudinal sinus, like little fungoid excrescences. When the dura mater was removed, the convolutions appeared flattened, from the profusion of fluid in the ventricles. The arachnoid was not very vascular, and there was no serous effusion.

On cutting into the substance of the brain, there were decided marks of congestion internally, and very distinct mottling. The corpus callosum was a little raised. The ventricles were distended to three times their natural size, by limpid fluid: the parietes and septum lucidum were firm: the foramen of Monro was large and open; one large vessel ran meandering along the under edge of the plexus choroides: the plexus itself was exsanguine.

The optic nerves were remarkably small, hard, and of a yellow colour, very different from the pure white by which they are usually distinguished. Their section was oval and compressed. The infundibulum was rather thicker, and of firmer consistence than natural.

Beneath the tentorium, a tumor, as large as a chesnut, was found on the left side, apparently attached by a peduncle to the petrous portion of the temporal bone, pushing aside the tuber annulare and the left hemisphere of the cerebellum, compressing the medulla oblongata, and pushing the fifth nerve upwards. This was found to be a firm dark tumor, the section of which was mottled with grumous blood; and it altogether bore the appearance of a fungoid growth, arising from the cancellated structure of the bone, but closely attached to the anterior portion of the cerebellum. The cancelli of the bone were soft, containing a puriform fluid. The tympanum was quite gone; and the ear contained some purulent matter.

In the lower part of the thorax, on the left side, was a circumscribed chronic empyema, of some standing.

The following observations by Dr. Bright are a good example of rational and supported generalization. It would be well, if all other observations in medicine were as rational and as supported.

"In both cases, we have individuals, little past the prime of life, dying in consequence of tumors similarly situated within the scull; and, as they were both in other respects healthy, their symptoms had suffered no important complications from the co-existence of other diseases. In both, we have reason to connect the aggravation, and probably the existence of the disease with the exposures and accidents of military service. In both, the disease has been marked by its gradual progress; has first shewn itself by affections of the senses; and then slowly produced paralysis of motion or sensation in various parts, affecting the intellect little, until an advanced period of the disease, and probably not before it had led to extensive serous effusion into the ventricles.

The symptoms may be more specifically stated; as, an almost total loss of sight, total loss of hearing in one ear, and to a great extent in both, gradual paralysis of the extremities, slight and temporary affection of the sphincters, great diminution in the sense of taste, and a protracted death from sensorial oppression.

In these two cases, the left ear lost its sensibility not much less than twenty years before death; in the one, from the concussion of a cannon; in the other, after a severe wound in the face, and, doubtless, concussion of the temporal bone. What predisposing influence was exercised by the violence inflicted at that time, in either case it is impossible to say; but the circumstance should not be lost sight of, in the record of facts.

In both cases, the vision was impaired and destroyed, even before the hearing of the right ear; and it is not easy to account for this affection of the sight. I am sorry that I cannot find any observation after death on the condition of the optic nerves in the first case; and I therefore suppose that no remarkable change was observable in their appearance, or that it was passed over unobserved. In the second case a very obvious alteration presented itself, the nerves being small, hard, and dark-coloured, with a yellow tint, and, to all appearance, unfitted for the discharge of their natural function; but how this change was induced, whether by pressure on any part of their course, or by interruption to the circulation through their substance, or by the irritation of contiguous parts, or in consequence of the serous effusion taking place in the ventricles, I do not pretend to say. The loss of vision, in both cases, leads us to suppose that it forms an important part of the consecutive history of the disease. The loss of sight in the left eye took place, in each, rather more than two years before death: and the loss of vision in the right eye followed very shortly after that in the left. The left ear, though its power was diminished to a very great extent in both cases, and was entirely lost in the first, retained its faculty of receiving impressions longer than either of the eyes.

The situation of the organic mischief, which might be said, in both cases, to encroach upon the mechanism of the right ear, and which made pressure on the auditory nerve of that side, afforded sufficient explanation of the destruction of its functions; but it is probably to the pressure communicated, through the pons Varolii, to the auditory nerve on the opposite side, as the tumours enlarged, that we may ascribe the slow dimunition of sense in the left ear.

With regard to the sense of taste, it seems to have been impaired, in each case, as the disease gradually advanced; and in neither is any specific notice taken of it, till a few months before death. This is one of the most peculiar and interesting symptoms because one of the least-frequently noticed in cases of cerebral lesion; and I have no doubt that it arose from pressure made by the tumour on the fifth pair of nerves, which gives origin to the gustatory branch; for, in both cases, the fifth pair suffered the most obvious and decided displacement and compression. The obtuseness of the sense displayed itself in the total want of preference with respect to articles taken into the mouth, as observed in the first case, so that the most nauseous medicines were taken with the same indifference as the most grateful beverages; and, in the second case, the inability to distinguish flavours was freely admitted."

B. *Lesions of the Spinal Cord.*

Dr. Bright relates four cases illustrative of the effects of pressure on the cord or of lesion of it.

CASE 1. *Paralysis of Motion from Pressure made by the Processus Dentatus.*

Samuel Elom, aged 52, admitted Aug. 13, 1832, having experienced stiffness of the neck for three months: and, after exposure to cold, two months before his admission, first suffered from loss of power in his left hand. Gradually, the right hand also became involved; then the left leg; and then the right leg; so that he now lay or sat in a perfectly helpless state, being only able to move very partially the right leg, which was occasionally raised in a convulsive manner, and the left leg also convulsed more slightly. The sensation was not impaired; the respiration was chiefly carried on by the diaphragm; and when he made a severe effort to cough, his legs were spasmodically drawn up. The muscles of the lower part of the face and the jaws appeared constrained. There was some difficulty in expelling the fæces and urine; but he was always able to retain them. He complained of some pain in the throat; and any motion of the head, so as to call either the atlas or the second vertebra into motion, produced considerable pain. A seton, the strict recumbent posture, and mild remedies were the means employed, with the object of procuring anchylosis. He suffered occasionally from violent fits of dyspnœa, which were relieved by stimulants.

In the middle of November, he began to recover the power of his right hand—towards the end of that month, he began to regain the use of the right foot —on the 8th of December, he could move the left leg a little—on the 11th of February, the ribs were considerably raised in inspiration—on the 28th, he had perceptible motion of the left hand. He continued to improve, and recovered so far that he could walk about without assistance. But in February, 1834, he got out of the Hospital, drank freely, and had his neck forcibly moved. The consequence was a relapse, and death in April.

Examination after death shewed extensive disease in the superior cervical vertebræ; and an abscess had lately formed, burrowing along the anterior surface of their bodies.

CASE 2. *Paralysis, with much spasmodic Affection of the Lower Extremities, from Disease in the Dorsal Vertebræ.*

James Carr, aged 28, a bookseller's assistant, exposed much to wet. His complaint began after such an exposure. The affection of the lower extremities began with weakness of them.

When admitted, he could walk a few steps, with the assistance of a couple of chairs; but even this exertion caused great fatigue, and excessive perspiration. The spinous process of the fifth dorsal vertebra projected: the two next below were depressed and tender, and yielding upon pressure. Below these, sensation imperfect, the numbness extending, in front, nearly up to the umbilicus: abdominal muscles, tense: twitching of the legs, which are drawn up: bowels confined.

Fiat Setaceum reg. dorsi.

Decoct. Aloës co. ℥vj. o. m.

Pil. Plum. gr. v. o. n.

During his stay in the Hospital, a period of seven months, he was sometimes better, sometimes worse; constantly tending, however, to an aggravation: sometimes sensation became more perfect: sometimes he could move the legs with greater facility: sometimes there was retention of urine; never incontinence; and he could always command the rectum. The urine was healthy. He left the Hospital at the commencement of January; and suffered severely from bronchitis, from which he is now recovering.

He has now complete loss of voluntary motion and sensation in the legs. The abdominal muscles have usually a board-like rigidity. Getting him out of bed brings on repeated spasms, attended with pain; and a hurried and laborious respiration, with a jerking expiration. The legs, when he is in the recumbent posture, are generally extended and crossed. He has perfect command over his rectum, and readily passes his urine, which is healthy.

The next case presents nothing to detain us. Indeed the two preceding cannot be looked upon as evincing any very extraordinary features. But they are fair samples of the effects of lesion of the cord, in two distinct situations, and we have given them in an abridged form.

c. *Lesions of the Brain.*

As Dr. Bright observes, the specific consequences of lesions of different portions of the brain are so little understood, that facts, noted carefully, are valuable.

CASE 1. *Difficulty of Articulation, and of connecting words with their corresponding ideas—Lesion in the Corpus Striatum*

Sarah Dodds, aged 30, admitted with hemiplegia of the right side, not affecting the face. Her articulation was exceedingly imperfect; but the most remarkable circumstance in her condition was, the total inability of connecting her words, when she could utter them, with the ideas she wished to express, or the things to which she meant to refer.

Her complaint had commenced seven or eight months previously with dyspepsia, headache, singing in the ears, &c. In the middle of June, that apoplectic seizure occurred of which hemiplegia was the result, and it was a little more than a fortnight after the seizure that she was admitted. Under a gently tonic plan of treatment, she improved in motion and intelligence. But the peculiar difficulty of connecting words with things remained.

"When asked, for instance, the name of her hand, at which I pointed, she said, 'A pin;' but immediately signified her knowledge that this was not the right word, though she could not tell what the right name was. On the 23d of September, at her own particular desire, she was permitted to have electricity, in the form of slight shocks and sparks, applied to the affected side, and her improvement seemed to be still further hastened under the use of this remedy; and, by the 1st of October, I noted her speech and articulation to be almost perfect, and that she connected words with ideas much better. She continued gradually to improve, and was able to walk, though with much effort, without a stick, along the whole ward. Her right hand, however, scarcely recovered at all, but remained stiff, with the fingers semi-flexed. Her powers varied; but in her best days she spoke like a foreigner, considering all her words, and not unfrequently mistaking them."

On the morning of the 24th of October, she had the premonitory symptoms of an apoplectic attack, which occurred that afternoon. She sank under it in three hours.

*Dissection.* Raising the calvaria and dura mater, the convolutions were seen compressed; and a fluid could at once be felt in the right hemisphere. Cutting into this hemisphere, it was found that a large portion of the cerebral matter was broken down and softened. There was an extensive yellow-ochery cell, shewing the situation of the injury which had attended the first apoplectic seizure; and this seemed to occupy the corpus striatum in all its extent, more particularly the posterior part, where it was surrounded by a thin fibrinous cyst, and contained a little brownish-yellow clot. The fatal attack seemed to have originated near the same part; to have burst, through softened brain, into the left lateral ventricle; completely destroyed the septum lucidum; filled the opposite ventricle; found its way into the third and fourth ventricle; and insinuated itself into the cerebellum; in the left hemisphere of which was a clot as large as a walnut, like black-currant jelly; while the blood lay in layers between the plates of the cerebellum, and formed an extensive ecchymosis under the arachnoid. The arteries were generally a little thickened, and spotted with atheromatous matter, evidently tending to ossification. The vertebral arteries were dilated.

Left ventricle of heart simply hypertrophied—mitral and aortic valves slightly thickened—ascending aorta rather dilated, and its coats thickened.

"In this case," says Dr. B., "the situation of the cerebral lesion left by the first attack was well defined; and, from its situation in the posterior part of the cerebral portion of the brain, it corresponded well with the opinion now pretty confidently received by many observers, that lesions of this portion are connected with a diminution of power in the arm; while its occupying chiefly the posterior part of the corpus striatum is further in accordance with an impression I have received from observation, and inculcated, with regard to the lesions which influence the articulation. As to the connexion of this particular lesion, and the peculiar mental difficulty, we can only say, that, as far as we can judge, the injury done to the corpus striatum corresponded, in point of time, with the commencement of the mental affection; and the mind was gradually regaining its power of associating words and ideas, while a process of gradual reparation was taking place in the lacerated brain."

CASE 2. Dr. Bright relates another case in which extreme difficulty of connecting words with ideas existed. The gentleman had once before had a fit—on this occasion had slight distortion of the mouth—but had recovered from the fit, and now recovered from this affection under depletion more or less active. We need not enter on the particulars of the case, as recovery prevented dissection, and the discovery of the lesion.

CASE 3. *Deranged Vision and Sense of Touch, with Lesion of the Optic Thalamus.*

In October, 1834, a gentleman, aged 57, had slight vertigo, fell, and, for several weeks afterwards, had numbness of the whole of his left side.

Two months after, he had another attack; which was followed by a very peculiar morbid sensation in his fingers, so that every thing he took hold of felt as if it were gelatinous or unctuous; and when he touched his bed-clothes, it was difficult to persuade him that arrow-root had not been spilt over them. This lasted for a short period, not many hours, and was succeeded by several optical delusions: he fancied he saw persons in the room, and that, as he walked the floor, he had to step over gates and stiles and railings, and his mode of stepping corresponded with this conviction; at the same time, he was capable of transacting business, so that the illusion appeared most probably to arise from some morbid impression of the optic nerve, and not from a mental process.

In the Autumn of 1835, he had dull pain over his ears, and indistinct hearing. In October, he suffered, for a few hours, an almost total loss of sight, and was, at the same time, unable to comprehend what was read to him; he always felt a tingling sensation in his left leg and thigh.

In the middle of the Summer of 1836, he was attacked with intense pain in the right hypochondrium, followed by inflammatory symptoms. He died in October, 1836.

*Dissection.* The sinuses much loaded: tunica arachnoides opaque, thickened, and raised by serous fluid, which flowed in considerable quantity on removing the brain, gravitating towards the basis cranii and vertebral canal.—Medullary structure unusually soft and vascular. On making a section of the posterior part of the right thalamus nervi optici (corpus geniculatum inferius), a chink or cell, half an inch in length, was detected on its surface, presenting a dusky yellow tinge, as though stained with bile; and there was increased softening of the surrounding medullary substance. Half an ounce of fluid in the right lateral ventricle: none in the left. The cerebral arteries were large and patulous, exhibiting occasional traces of atheromatous deposit.

The connexion between impaired and disordered vision and lesion in the optic thalamus is obvious, and, in reference to our physiological and pathological opinions, satisfactory.

---

Royal Naval Hospital, Plymouth.

Case of Gastro-Enteritis, and Phlebitis Portalis. By Sir David J. H. Dickson, Knt. M.D.

Gentlemen,—The following outline of an extraordinary case of gastro-enteritis, and phlebitis of the portal system, I have no doubt will be deemed well worthy of record in your valuable Journal.

William Cleves, Royal Marine, æt. 26, was admitted into this hospital with symptoms of gastro-enteritis, on the 21st February, 1837. He had embarked on board the Scorpion Brig, just commissioned, on the 18th, and said that he was taken ill the same evening: but having been on leave, and drinking for several days previously, he did not apply for medical aid until the morning of the 21st, when he was blooded, and immediately sent to the hospital. He afterwards stated that he had been ill for four days; but except from his own account, which could not be relied on, and what he told the nurse, little of this man's history was known until after his decease; when I learned from one of his former shipmates, that he was considered an idle dissipated character; that he had been subjected to corporal punishment, in the early part of December, while serving in the Cornwallis, in the Tagus, for refusing knapsack drill, and to obey the orders of the serjeant; and that for this, and other acts of insubordination, afterwards, he had been ordered to walk about for a certain time daily, with his knapsack and accoutrements buckled on:—the pressure of which, I believe, as in other kinds of "tight-lacing," has been productive of injurious effects, on many occasions, by inducing or aggravating gastric, and thoracic, but especially cardiac disease. On the passage homewards this man appeared to be unwell occasionally, but even by his messmates was believed to be malingering; and on the arrival of the ship, he was affected with the then epidemic catarrh: but I have no doubt that he concealed his illness, when disembarked at head-quarters, for the purpose of obtaining leave, and indulging in those intemperate habits to which he was so much addicted.

Upon admission, this patient, who was of a delicate nervous temperament, complained of pain, a sense of weight and heat, and great tenderness, on pressure, in the epigastric and abdominal regions; with a weak sharp pulse, tongue red, and furred in the centre; great anxiety, and depression, frequent vomiting, and other symptoms of gastro-enteritis. The treatment consisted of general and local depletion, fomentations, gentle aperients, and alteratives, enemata, effervescing and sedative medicines, to allay the gastric irritability, and blistering, &c. By these measures he appeared for a while to be relieved, and the disease assumed more of an hysterical, and intermittent character; so much so, that a trial was made of the sulphate of quinine; but it disagreed with him, and was immediately relinquished. He generally shed tears when spoken to; complained of pain in his limbs, which were often agitated by convulsive motions, and in every part of the body, in succession; and he had, also, frequent rigors, followed by the hot and sweating stages; but except during the exacerbations, when it was much quickened, the pulse was usually about 84, from which it ranged to 96, and 100 beats in a minute; but unequal in strength, and frequently intermitting.

It would be impossible, however, to give an intelligible account of the ever-varying symptoms—the patient, at one time, declaring that he was very ill; at another, that he was much better, or quite well; particularly after an anodyne, which immediately allayed the nervous irritability. Indeed, as the case advanced, the symptoms became so anomalous, variable, and complicated by their ubiquity, if I may use the expression, and by occasional delirium, as to involve the diagnosis in the greatest obscurity. It was evident that there was much mischief going on; but such extent of disease could scarcely have been appreciated, until revealed by dissection. Latterly, however, the obscurity cleared away—and the swelling of the abdomen—the great prostration of vital power—the rapidly increasing emaciation—the frequent rigors, terminating in profuse sweating—and other symptoms of hectic, unequivocally denoted the existence of internal suppuration—and he died on the 16th of March, 1837.

Without indulging in speculations, for which I have neither leisure, nor you room, probably, at present, upon the pathological appearances about to be related,—or entering upon the questions of the primary, or secondary nature of the phlebitis,—or the connexion between inflammation of the inner venous membrane, and secondary affections, with purulent deposits—questions which have been anxiously discussed by the first practical authorities, and frequently and ably adverted to in your Journal, I now proceed simply to detail, without comment, the morbid appearances observed on dissection, in the interesting case under consideration.

*Sectio Cadaveris* 14 hours, post mortem. There were slight adhesions between the pulmonary and costal pleura, on the left side of the chest; but the lungs and the heart were in a healthy state. The abdominal cavity contained about three pints of yellowish turbid serum, intermixed with flocculi of lymph and albumen. The peritoneal surface of the intestines was of a dark cineritious colour; but, in some places, mottled and rough, from the effusion of coagulating lymph; by which exudation different portions of the intestinal convolutions were agglutinated together, and to the contiguous viscera. The opposite portions of the parietal peritoneum, also, exhibited a reddish, mottled, or ecchymosed appearance. The stomach was distended with gas and fluid; and its coats were so softened as to be easily lacerated. About an inch and a half below, and to the right of the cardiac extremity, a purulent exudation was observed, from a small opening externally, which descended by a sinuous passage into the stomach: the superior situation, and obliquity of which would, by acting like a valve, prevent any escape of its contents. The subserous cellular tissue was in many places infiltrated with a purulent looking fluid. On removing this organ, and nearly opposite to the above-mentioned aperture, the posterior surface of the stomach was found abraded by ulcerative inflammation, and the cellular tissue between it and the pancreas was loaded with purulent deposition. Upon opening the stomach, it was found to contain a large quantity of gas, and of dark green fluid, such as he had vomited before death; and its mucous surface was so soft, that it could be readily scraped off with the nail. The pancreas was much indurated, and by its removal the splenic vein behind, which was intimately attached to it, was ruptured; from which flowed a quantity of purulent matter of a deep yellow colour. This extraordinary appearance led to a careful examination of the portal system generally, and its ramifications in the liver and spleen were attentively traced by my first assistant, Mr. Weale, who is an excellent anatomist. They were found, throughout, to retain a similar puruloid fluid; but in those of the liver it seemed to be mixed with thick bile; and in many of the vessels there were somewhat long, but slender coagula of blood. The lining membrane of the larger trunks was rendered rough by filmy attachments of lymph; and their coats were thickened, and preserved a cylindrical form; so that, on a superficial examination, they might have been mistaken for arteries. The liver was soft, and the spleen, also, normal; but both afforded a similar puruloid exudation on pressure, — it may be presumed, from their minute venous ramifications. The venæ cavæ hepaticæ, and the mesenteric veins were also, perfectly healthy. The gall-bladder contained about two ounces of greenish bile, and the ducts were pervious. On detaching the caput coli, a large collection of pus was found deposited in the cellular tissue, between it and the iliac muscles: and the ulcerative process had destroyed the muscular and mucous tunics, causing a small opening into the gut; but the direct apposition of the intestine to the iliac wall, prevented the escape of any of its contents by the perforation. The internal surface of the intestines, and the mesenteric glands were natural.— The structure of the kidneys was softened, but otherwise normal; and the urinary bladder did not present any unusual appearance.

DAVID J. H. DICKSON.

P.S. I have at present a patient labouring under ascites, who has been repeatedly tapped; and, latterly, 26 pints of fluid have been abstracted every eight or ten days. He has now been relieved by paracentesis 12 times; and how much longer this may go on is uncertain: but as there is much visceral disease, I cannot expect the same felicitous result as in the extraordinary case of the officer who recovered after having been, also, tapped 12 times, four years ago. He remained quite well the last time I heard of him.

---

The preceding case is an interesting one, and we feel much indebted to our able friend, Sir David Dickson, for enabling us to present it to our readers.

The history and the circumstances of the case would rather tend, we imagine, to this:—that inflammatory action, originating in the gastro-intestinal mucous membrane, extended, by contiguity of tissue, to the serous and the cellular membrane — and that the phlebitis was consequent on the inflammation and suppuration which ensued.

This explanation of the facts is more consistent with the history of the case and with what occurs after injuries, than the supposition that the phlebitis was the primary affection. The case itself will repay an attentive perusal.—*Editors.*

---

### Birmingham Eye Infirmary.

Report of the Cases attended by R. Middlemore, Esq. from Jan. 1, to December 31, 1836.

There are few who pursue the practice of ophthalmic surgery with more zeal than Mr. Middlemore, of Birmingham. His various contributions to Journals and Transactions evince both his industry and his information in this particular department of our science.

The following Report is contained in the Transactions of the Provincial Medical and Surgical Association, published within these last few days. The cases attended by Mr. Middlemore in the year 1836, are:—

Simple acute conjunctivitis, 116. Chronic conjunctivitis, 45. Acute conjunctivitis, with pustule or ulcer on the cornea or conjunctiva, 98. Acute conjunctivitis with puriform secretion, 55. Purulent conjunctivitis of new-born infants, 45. Irritable conjunctivitis, 53. Strumous conjunctivitis, 46. Effusion of various kinds beneath the conjunctiva, 13. Disease of the semilunar membrane and lachrymal caruncle, 4. Corneitis, 26. Vascularity of the cornea, 7. Pannus, 4. Opacity of the cornea, 44. Staphyloma of different kinds, and of various parts, 13. Ossification of the cornea, 1. Impaction of foreign bodies in the cornea and conjunctiva, 22. Sclerotitis, 13. Ulceration of the sclerotica, 3. Morbid growth from the cornea and sclerotica, 2. Pterygium, 3. Simple acute iritis, with or without ulcer of the cornea, onyx, or hypopium, 34. Chronic iritis, 3. Syphilitic iritis, 3, Strumous iritis, 5. Prolapse of the iris, 4. Vacillation of the iris, 3. Choroiditis, 6. Varicose ophthalmia, 3. Retinitis, 2. Cataract, 29. Fungoid and other tumours within, and upon the surface of, the eye-ball, 7. Amaurosis of various kinds and in different degrees, 50. Diseases of the lachrymal apparatus, 21. Strabismus, 7. Ophthalmia tarsi, 71. Lippitudo, 7. Hordeolum, 4. Entropium, 5. Ectropium, 3. Inflammation of the eye-lids, 12. Ptosis, 1. Ulceration of the eye-lids, 6. Tumours in the eye-lids, 20. Wounds of the eye and its appendages, 30. Adhesion of the eye-lid to the eye-ball, 4. Congenital defects, 6.

Mr. Middlemore notices more circumstantially some particular affections.

### 1. *Irritable Ophthalmia.*

"In three examples of irritable ophthalmia, which have fallen under my observation during the past year, and which occurred in poor women, who had continued to suckle for an injudiciously long period, in the hope of deferring their subsequent pregnancy, one eye only was affected in each case; and, on inquiry, I found that the subjects of the malady suckled only with the breast of the side corresponding to the diseased eye. I do not know a more decided proof of the asserted connexion subsisting between protracted lactation and the irritable form of ophthalmia, which occasionally affects the eyes of females during that period."

We do not know that this can be considered *very* decided evidence of the connexion between lactation and ophthalmia. The best evidence is the frequent coincidence of the two affections, such frequency of coincidence or of sequence constituting, in fact, the ordinary evidence of cause and effect, or of the relations of one thing to another.

### 2. *Carcinomatous Growth from the Cornea and Sclerotica.*

Joseph Wilkes, æt. 49, residing at Redditch, has been suffering for several weeks from a painful tumour arising from the surface of the eye It is about the size of a shilling, of a red colour, firm texture, and very uneven surface; it is firmly attached to the surface of the sclerotica and cornea, but chiefly to the former; it is not very painful when touched. The conjunctiva is inflamed, but the cornea and iris are not so affected. There is very copious lachrymation, and considerable intolerance of light. The patient is employed as a waggoner; and he states that the disease has come on gradually, and from no cause with which he is acquainted.

Mr. Middlemore was desirous of removing the whole anterior part of the eye-ball. He was over-ruled, but no doubt, he was right. He dissected away the morbid growth from the surface of the membranes to which it adhered. In about two months after the patient's return home, the growth re-appeared, and has since rapidly increased. But the patient will not submit to another operation.

The growth, when examined, was found to be hard, and interspersed with firm white bands and spots; its surface was vascular, and had a rough craggy appearance. It more nearly resembled cancer of the eye-ball than any other disease.

3. *Melanotic Growth from the Semilunar Membrane.*

"I have now a patient in attendance at the Eye Infirmary, in whom there is a filmy prolongation, of a melanotic character, of the membrana semilunaris; and, just at the temporal side of the lachrymal caruncle, there is also a dark-coloured tumour of the size of a large pea. It appears, though much darker, a good deal like a portion of iris recently prolapsed through an opening in the cornea. The disease has existed for several years, but has lately much increased owing to her having scratched it whilst rubbing her eye. I have told this poor woman that nothing but a surgical operation is likely to relieve her, but she will not, at present allow me to remove the diseased part. I have never before seen a case precisely similar to this, during the whole course of my practice.

Since the preceding paragraph was written the tumour increased in size so very rapidly that the patient was anxious to have it removed. This was readily accomplished by means of the small curved scissors, as it was but slightly adherent to the semilunar membrane, and quite unattached to the sclerotica. It was covered by the conjunctiva, and appeared to have been chiefly developed in the subconjunctival cellular membrane. The tumour itself was not organized: it consisted of a firm substance of an irregular dark colour, its external surface being densely black, its middle and posterior portions being of a dark brown colour, intermixed with lines and streaks of an extremely black appearance. The whole of the tumour became comparatively pale after it had been immersed in spirit of wine three or four days."

4. *Ossification of the Cornea.*

Job Woodcock, æt. 69, had suffered for some months from extreme pain in the left, and serious irritation in the right eye—he is troubled with chronic rheumatism.

The right eye appears to be suffering only in consequence of the disease in the left. On a careful examination of the left eye, it was ascertained that the cornea was the seat of ossific deposition at several points; that the lamellæ, beneath which one of them existed, had become absorbed; and that the friction of the sensitive lining of the palpebra upon this partially exposed portion of ossific matter occasioned the whole of his uneasiness.

With an ordinary extraction-knife, Mr. M. made a semi-circular incision at the lower part of the cornea, just beneath the last ossific deposition; then, raising the flap with the forceps, he rapidly detached its superior part. In a day or two, the man could resume his occupation.

5. *Circumscribed Vascular Prominence of the Cornea.*

Edward Newton, æt. 13, of a scrofulous constitution, has a prominence of considerable size at the fronto-temporal part of the sclerotica, which has existed for several weeks. The prominent part is very vascular and full of large pink-coloured vessels: it does not appear as though the texture of the sclerotica were either attenuated or thickened. The part is not particularly tense or tender when touched. The disease came on gradually, without local injury. It is not attended with pain, and scarcely at all affects the sight.

No medicines were of any service. Mr. Middlemore has seen other cases, which were equally irremediable. The projections, after attaining a certain magnitude, do not appear to increase.

6. *Extensive Bulging of the Sclerotica.*

Louisa Chatwin, æt. 59, of a feeble constitution, has suffered on various occasions from acute rheumatism. The right eye became tender and dim, but without much pain or redness, many months ago, and the sight of it now is nearly destroyed.

*State of the Eye.*—The cornea is cloudy, and studded with a considerable number of very minute ulcers. The pupil has a glaucomatous appearance, and the sclerotica is considerably bulged at its temporal side. The enlarged part of the sclerotica is slightly discoloured, but it is not very vascular. The mouth was made sore by the administration of mercury, and with the effect of removing the diseased appearance of the cornea.

7. *Disease somewhat resembling Malignant Affection of the Eye-ball.*

G. S. æt. 10, a healthy boy, complained of uneasiness of the right eye, with loss of vision, a few days before he applied to Mr. M.

"*State of the Eye.*—There is a very faint arrangement of pink vessels around the cornea, and slightly increased lachrymation. The iris is pushed towards the cornea so as to be nearly in contact with it, and to resemble a small short funnel with its broad part directed towards the retina. The pupil exhibits a bright shining appearance, exactly like what I have seen in cases of incipient fungus hæmatodes, but there are no blood-vessels to be seen ramifying within the eye. The pupil is not very small. The lens appears to be slightly cloudy, and to be in contact with the iris, except at its most central point, with which, indeed, none but an almost fluid lens could so adapt itself as to be in actual contact with it at that part. On his next visit it was observed that the pupil was nearly closed—it was a mere perpendicular fissure, pretty much like that of the cat when the eye is exposed to a bright light. The pupillary margin is drawn backwards, so as to render the iris convex, just as though, in its expanded state (contracted pupil), its pupillary and ciliary margins being fixed, a solid body was pushed against its intervening part. During the last few months the eye appears to have undergone no change, except that it is entirely free from uneasiness, and is slightly atrophied. When I last saw him the eye appeared to be still more atrophied, but in other respects, pretty much as before. The opposite eye is quite strong and perfect."

Mr. M. employed a course of mercury. He has witnessed several cases very similar to the preceding, which terminated in atrophy of the eye-ball.

---

### WORCESTER INFIRMARY.

REPORT FOR THE YEARS 1835 AND 1836.*

In the year 1835, there were admitted into the Infirmary, as in-patients, 666 patients; 28 of whom died.

The following tables shew the number of cases, and of deaths, which occurred in each month, distinguishing those under the age of 20 years, from those above that age, and also whether males or females:—

* Provincial Transactions.

| | MALES. | | | | FEMALES. | | | |
|---|---|---|---|---|---|---|---|---|
| | Under 20 years. | | Above that age. | | Under 20 years. | | Above that age. | |
| | CASES. | DIED. | CASES. | DIED. | CASES. | DIED. | CASES. | DIED. |
| January.... | 5 .. | 0 | 29 .. | 2 | 10 .. | 0 | 26 .. | 2 |
| February..... | 6 .. | 0 | 30 .. | 3 | 6 .. | 0 | 17 .. | 0 |
| March ...... | 6 .. | 0 | 30 .. | 2 | 8 .. | 0 | 17 .. | 0 |
| April........ | 7 .. | 0 | 27 .. | 2 | 10 .. | 0 | 8 .. | 1 |
| May ........ | 8 .. | 1 | 42 .. | 3 | 11 .. | 0 | 11 .. | 0 |
| June ........ | 9 .. | 0 | 26 .. | 1 | 8 .. | 0 | 8 .. | 1 |
| July ........ | 11 .. | 0 | 26 .. | 1 | 2 .. | 0 | 15 .. | 1 |
| August ...... | 3 .. | 0 | 30 .. | 1 | 9 .. | 0 | 25 . | 0 |
| September.... | 7 .. | 1 | 22 .. | 0 | 5 .. | 0 | 8 .. | 0 |
| October...... | 5 .. | 0 | 20 .. | 1 | 4 .. | 0 | 15 .. | 1 |
| November.... | 6 .. | 0 | 30 .. | 1 | 9 .. | 2 | 14 .. | 0 |
| December .... | 2 .. | 0 | 20 .. | 0 | 8 .. | 0 | 5 .. | 0 |
| Total .... | 75 | 2 | 332 | 18 | 90 | 2 | 169 | 6 |

| | TOTAL MALES. | | TOTAL FEMALES. | | OF BOTH SEXES. | |
|---|---|---|---|---|---|---|
| | CASES. | DIED. | CASES. | DIED. | CASES. | DIED. |
| January ...... | 34 .. | 2 | 36 .. | 2 | 70 .. | 4 |
| February...... | 36 .. | 3 | 23 .. | 0 | 59 .. | 3 |
| March........ | 36 .. | 2 | 25 .. | 0 | 61 .. | 2 |
| April ........ | 34 .. | 2 | 18 .. | 1 | 52 .. | 3 |
| May.......... | 50 .. | 4 | 22 .. | 0 | 72 .. | 4 |
| June ......... | 35 .. | 1 | 16 .. | 1 | 51 .. | 2 |
| July.......... | 37 .. | 1 | 17 .. | 1 | 54 .. | 2 |
| August ....... | 33 .. | 1 | 34 .. | 0 | 67 .. | 1 |
| September..... | 29 .. | 2 | 13 .. | 0 | 42 .. | 2 |
| October ...... | 25 .. | 1 | 19 .. | 1 | 44 .. | 2 |
| November .. . | 36 .. | 1 | 23 .. | 2 | 59 .. | 2 |
| December ..... | 22 .. | 0 | 13 .. | 0 | 35 .. | 0 |
| | 407 | 20 | 259 | 8 | 666 | 28 |

The proportionate mortality in males and females, under 20 years of age and over it, is displayed in the next Table.

| | Under 20 years. | | Died. | | Proportionate mortality. |
|---|---|---|---|---|---|
| Males........ | 75 | .. | 2 | .. | 1 in 37,5 |
| Females ...... | 90 | .. | 2 | .. | 1 in 45, |
| | 165 | | 4 | .. | 1 in 41,25 |
| | Above that age. | | | | |
| Males ....... | 332 | .. | 18 | .. | 1 in 18,44 |
| Females...... | 169 | .. | 6 | . | 1 in 28,166 |
| | 501 | | 24 | .. | 1 in 20,875 |
| | Males. | | | | |
| Under 20 years. | 75 | .. | 2 | .. | 1 in 37,5 |
| Above that age. | 332 | .. | 18 | .. | 1 in 18,44 |
| | 407 | | 20 | .. | 1 in 20,35 |
| | Females. | | | | |
| Under 20 years. | 90 | .. | 2 | .. | 1 in 45, |
| Above that age. | 169 | .. | 6 | .. | 1 in 28,166 |
| | 259 | .. | 8 | .. | 1 in 32,375 |

| | Total. | | | | |
|---|---|---|---|---|---|
| Males ........ | 407 | .. | 20 | .. | 1 in 20,35 |
| Females ...... | 259 | .. | 8 | .. | 1 in 32,375 |
| | 666 | | 28 | .. | 1 in 32,78 |

In the year 1836, there were admitted as in-patients 618 persons, 37 of whom died. Of the 618 admitted, 374 were males, and 244 were females. In the following Table, we see the number of cases which occurred in each month, and distinguish the patients under and over 20 years of age, as well as the males and females.

| | MALES. | | | FEMALES. | | |
|---|---|---|---|---|---|---|
| | Under 20 yrs. Cases. | | Above that age. Cases. | Under 20 yrs. Cases. | | Above that age. Cases. |
| January...... | 6 | — | 28 | 10 | — | 16 |
| February .... | 5 | — | 21 | 6 | — | 15 |
| March ...... | 7 | — | 24 | 8 | — | 12 |
| April........ | 7 | — | 28 | 3 | — | 16 |
| May ........ | 2 | — | 14 | 2 | — | 11 |
| June ...... . | 7 | — | 19 | 7 | — | 18 |
| July ........ | 7 | — | 34 | 8 | — | 12 |
| August ...... | 5 | — | 32 | 6 | — | 18 |
| September.... | 3 | — | 31 | 10 | — | 9 |
| October ..... | 7 | — | 29 | 7 | — | 14 |
| November.... | 5 | — | 21 | 5 | — | 12 |
| December .... | 6 | — | 26 | 6 | — | 13 |
| | 67 | | 307 | 78 | | 106 |

### CLINICAL FACTS AND OBSERVATIONS.

We have thought that it might be as well to collect together some of the many valuable hints, that we find in reports of Clinical Lectures and of Hospital cases. They may form a sort of tail to the more elaborate articles which have gone before.

1. *Opening a small Abscess.*
" If you consult those books which treat of abscess, you will find it laid down as a general rule, that where the abscess is of small size, it ought to be left to nature to effect an opening, because this, it is said, will be small, and consequently leave but an inconsiderable cicatrix. According to this view, small abscesses are to be left to themselves, provided they be not too indolent, nor advance too rapidly. But I reject this method; for if the aperture made by nature be small, why should not that made by art be made small likewise? It is only necessary for this purpose that we use an instrument with a narrow blade, and that we make a simple puncture."— *Lisfranc, Med. Gaz.*

2. *To prevent Scars in the Neck.*
" Abscesses of the neck ought to be opened by means of a simple puncture. I do not now allude merely to small abscesses: I have opened, in this manner, purulent depôts of considerable size, and, although the extent of the incision was not in proportion to the collection of matter, yet was all the pus evacuated, while the cicatrix which remained did not exceed that of a leech-bite. This precept is of great importance, not only to the welfare of the patient, but to the reputation of the surgeon."

If there is a lodgement at the lower part, a small counter-opening is necessary. The incision, in any case, should always be made transversely, in the direction of the natural folds of the skin.—*Ibid.*

"3. *How to open a large Abscess.*

"The bistoury is to be held in the first position: the two last fingers, separated from each other, and extended, are to be placed, if possible, beyond the tumour, as a *point d'appui:* the tissues which are penetrated must be divided in a perpendicular direction: the middle finger, placed on the blade of the instrument, serves to regulate the depth of the incision. This is very important, for if the instrument cuts ill, or if the texture be hard, we are under the necessity of pressing more strongly on the parts to be divided; and without the precaution of having the finger as I have described, we should incur the risk of plunging in the instrument too far. Besides, it is easy to push the bistoury father in if necessary, by drawing back the finger on the blade of the instrument. We must do all gently: thus, when the blade arrives in the collection of pus, the hand will perceive the fact, because the knife is now passing through a less resistance than before. The only exception to this is where there are muscular contractions of a nature to interfere with the resistance. I cannot well give you a measure of the slowness necessary in this proceeding; but always remember this fundamental principle in operative surgery—*tuto* is better than *cito.*"—*Ibid.*

It is refreshing to hear a man of the admitted dexterity of Lisfranc, of a dexterity probably quite unequalled, denouncing the stop-watch mode of operating. A master of celerity himself, he points out the folly, we might almost say the wickedness, of making it the paramount consideration.

4. *How to open a deep Abscess in the Neck.*

"Take the neck as an example: I there make an incision parallel to its axis, and which divides layer by layer successively the skin, the cellular membrane, and, if necessary, the superficial aponeurosis. I next take a blunt probe, and limit the extent to which it is to penetrate the textures, by holding it between the thumb and fore-finger. I then introduce this to the bottom of my incision, and make it pass on by separating, or rather pushing aside the fibres of the parts beneath. Whenever the instrument has entered the abscess, there is a cessation of resistance, besides which I perceive drops of pus oozing along the sides of the instrument. I then push it upwards and downwards, so as to enlarge the opening, and thus the matter finds a ready exit."—*Ibid.*

5. *Uncertainty of Dividing the Stricture in Hernia, external to the Sac.*

This case is related by Mr. Adams in Medical Gazette.* It deserves attention, because it shews the danger that *might* result, from the operation of dividing the stricture in hernia, without opening the sac.

March 14.—A man, 39 years of age, was admitted into the London Hospital with the usual symptoms of strangulated hernia. He had been the subject of inguinal hernia for many years, but had never worn a truss, and latterly the hernia had been irreducible. Two days ago a fresh protrusion took place, which he was unable to return. His bowels had been moved on the same morning. The ordinary symptoms of strangulation set in, and he was brought to the hospital in a state of much depression. Very slight attempts at reduction were consequently made, which were unsuccessful, and the operation was performed. The sac contained no fluid; a large portion of omentum was observed, of a healthy appearance. On unfolding this, a small *knuckle* of intestine was seen; this was found to be tightly strictured in the vicinity, apparently, of the inner ring. The intestine could be easily pushed towards the abdomen, but immediately returned. The stricture was divided, and the intestine readily passed into the abdomen. There was a distinct annular line indicating the situation

* April 22, 1837.

of the neck of the original peritoneal sac, but this was much below the strangulated gut. The man died on the following morning, apparently from the mere impression upon his constitution, for the intestine is in a very favourable condition. On making an examination after death, it became apparent that a portion of intestine had descended through an opening formed by a band stretching across from the omentum on one side to a portion of adjacent intestine, and in this had become strangulated, and consequently the stricture in this instance had been divided within the abdominal cavity."

We quite agree with Mr. Adams, that the stricture in hernia very often results from an altered condition of the narrow portion, or what may be termed the mouth of the sac, which in that situation becomes exceedingly thickened.

6. *Why the Recurrent Nerve is Recurrent.*

The course of the recurrent nerve is explicable without referring to any particular functions it could be supposed to favour. Tiedemann has shewn that in the short neck which the early embryo possesses, the first branch which the aorta gives off arises opposite the lower margin of the face, and therefore almost on a level with the origin of the recurrent nerve, which at this time passes straight to the larynx. But as the neck elongates, the subclavian arteries descend, and bring down the nerve with them. This is confirmed by those rare cases of anormal arrangements in which the right subclavian, arising on the left side of the chest, passes across the œsophagus to the other side; for in these the inferior laryngeal nerve of the right side has not a recurrent course, but passes straight in the larynx, as shewn in a preparation of the museum of the College of Surgeons in Dublin, of which a drawing was exhibited. Other instances of a recurrent course of nerves are found in the dental nerves of porcupines and other rodentia, as shewn in a specimen where the filament supplying the incisor tooth, arising in the middle of the dental canal, is obliged to return at an acute angle to reach the pulp of the tooth.*

These observations are extracted from an interesting Lecture delivered at the Royal College of Surgeons, by that able anatomist, Mr. Stanley.

7. *Cause of the Inclusion of the Tendon of the Biceps in the Scapulo-humeral Capsule.*

The tendon of the biceps is in the early embryo altogether outside the shoulder-joint, but in succeeding periods becoming, as it were, protruded into the cavity, it carries upon and after it a duplicature of the synovial membrane, which attaches it to the upper wall like a mesentery. But this double fold soon splits and disappears, and the tendon is left, passing through the cavity of the joint, surrounded by synovial membrane, which is continuous with that lining the capsular ligament only at its two extremities.†

8. *Expense of Patients in the Royal Infirmary of Glasgow.*

The expense of each patient, taken in its fullest extent, comprehends his equal share of the whole ordinary expenditure—that is, repairs, furniture, and incidental charges, as well as salaries, medicines, and provisions. This entire sum, amounting during the year to £6135. 6*s.* 8*d.*, divided by 5130, the whole patients, dismissed or dead, gives £1. 3*s.* 11*d.* the average sum which the treatment and maintenance of each patient, one with another, costs the establishment. Otherwise if the above sum, £6135. 6*s.* 8*d.*, be divided by 327, the average number of beds occupied throughout the year, the quotient £18. 15*s.* 3*d.* will be the expense of each bed constantly occupied for the whole year with a succession of patients. If we restrict the expenditure to the salaries, medicines, and provisions, leaving out the repairs, furniture and incidents, the amount now reduced to £4957. 8*s.* 6*d.* and divided

---

* Med. Gaz. June 3, 1837.

† Ibid.

as above stated, will give 19*s*. 4*d*. for the net expense of each patient, and £15. 3*s*. 2*d*. for the annual charge attending the continued occupation of each bed. If we confine ourselves to provisions alone, the expenditure, thus farther restricted, being £3479. 6*s*. 6*d*., and subjected to the same calculation, will give 13*s*. 7*d*. for the average cost of provisions furnished to each patient, and £10 12*s*. 9*d*. as the sum required to supply provisions to all the occupants in succession of each bed for the whole year.*

9. *Gouty Ramollissement of the Spinal Marrow.*

Professor Graves, in his valuable Clinical Lectures, alludes to this affection, which he believes not to have been hitherto described.

"It has been long known that gout may attack the brain, and the existence of gouty paraplegia is well known by practitioners who have studied attentively the progress of arthritic affections. Thus, in a case which I witnessed some time back, in consultation with Mr. Kirby, he prognosed the supervention of paraplegia at a time when the indications of its approach could not have been discovered by an observer of less experience and sagacity. I have already stated that gouty affections of the brain have long been known, and I am not sure that some of the older authors may not have alluded to gouty affections of the spinal marrow; but as our knowledge of the peculiar state of the brain and spinal cord, termed ramollissement, is comparatively recent, and not dating with any degree of accuracy earlier than the works of Abercrombie, Rostan, and other modern authors, it is obvious than any observations made by the older writers, concerning gouty affections of the nervous centres, can have no distinct reference to this lesion. The connexion, therefore, of ramollissement of the spinal cord with gout, may be considered as now, for the first time, distinctly pointed out."

Two cases are related in illustration.

* Annals of Medicine.

We will abridge the particulars of one, as a sample of the nature of the malady; it is, at all events, a good case of softening of the medulla.

*Case.* Mr. —— was much addicted to field sports, and would wade up to his middle in water, for many hours, during very cold weather. He was abstemious in his habits. In 1825, when about 25 years of age, he had fever, attended with inflammation of the joints, and said to be rheumatic; some pain and stiffness, and an evident enlargement of the knee-joints, remained, after the other articular affections had disappeared; these symptoms, however, yielded, in a few months, to rest and appropriate treatment. In the Spring of 1832 he was attacked with pain in one foot, supposed to be of a gouty nature: this pain disappeared during a drive of fifteen miles in an open carriage, but a certain degree of tenderness remained, and was always felt, more or less, in the part originally affected. He had a similar attack and pain and tenderness in the same foot in the following Autumn. This too diminished during a drive of twenty miles. In August 1833, he had a similar, but much more severe attack; the pain was much more violent than before, and both feet were affected. This, however, did not prevent him from following field sports as usual; he went on horseback to the mountains to shoot grouse, and to this exercise, and drinking a bottle of wine, he attributed his speedy, or rather sudden recovery from the pain in his feet.

In September 1833, he was seized with violent colic, attended with singular restlessness, and with singultus, and succeeded by jaundice. In January, 1834, he had another attack of colic, preceded by a fit, and similar attacks recurred frequently until August, 1835, when tremor of the fingers became visible. During some preceding attacks, he used to complain of weakness of the wrists and pains in the fingers, particularly the last joints. As the disease progressed, these pains became more intense and extensive, and the torture he felt in the hands and arms was beyond description. After August 1835, he began to lose the use of his arms, the tremors increased, and he began to complain of stiffness about the neck, with great restlessness and anxiety. The abdominal attacks came on occasionally, but not so severely as before. The arms became gradually weaker, until the loss of muscular power was complete, and they were greatly emaciated. The intellectual faculties remained perfectly unimpaired. In October 1835, two months after the state of the upper extremities had indicated the approach of paralysis, the lower extremities became similarly engaged: they were affected with tremors and weakness, and in the following December the patient had an attack of violent pain, with swelling and increased heat in the ball of one foot, which was pronounced to be of a distinct gouty character. After each attack of pain in the feet, the loss of power in all his limbs increased, and he was always rendered worse. All medical and other advice was useless, and in October, 1836, he died, having, for some time previously been greatly emaciated, quite paralytic in all his limbs, but not affected with any impairment of his intellectual powers.

*Dissection.* Dr. Graves was informed by Dr. Williams, who conducted the examination of the body that the viscera of the thorax and abdomen were healthy and normal, that no derangement or lesion of the brain could be detected, but that the spinal cord opposite to the last cervical and first dorsal vertebræ was softened to the consistence of thick cream; the remainder of the cord was also softer than natural, but did not present any thing peculiar in other respects.

Nothing, we imagine, can be more reasonable, nothing more consistent with the facts of the case, than the supposition that this gentleman's imprudent habits, so favourable to the occurrence of rheumatic inflammation, so calculated to repel gout from the extremities and surface, were the means of setting up inflammatory action of a gouty nature in the spinal cord. But it must be owned that in this, and in other instances, gouty inflammation must be received in a latitudinarian sense, and that strict induction would limit us to the admission *that* inflammatory lesions occurred in such or such an organ, in a gouty habit, under circumstances calculated for the retrocession of gout from the surface.

10. *Reformation of Factory Morals.* "Independently of the present voluptuous, I may almost say, indecent, fashion of female dress, which to our national disgrace, is only seen in these kingdoms; the heat of the workshops compels the young women and girls, who work in them, to dress both lightly and loosely, which cannot fail to inflame the passions of their male fellow-labourers. And even, although there may be a separation of the sexes, no modest person can enter either department without having their ears offended by oaths and indecent language. The remedy appears to me to be simple and easy, viz., by making the older and steadier persons, both male and female, monitors to the rest, who in order to remove the inquisitional appearance of the system, should be stimulated by suitable rewards, to imitate their good conduct, and to try to obtain a similar appointment, to which a higher rate of wages should be attached. Thus, at the expense of a few shillings per week, the manufacturer would have the satisfaction of knowing that for his private gain he had put no stumbling blocks in their way, nor had 'led' any person 'into temptation;' and would, eventually, derive a greater advantage from the labour of his dependents; for it is an invariable rule that the steadiest workpeople are the most gainful to their employers."

These observations are contained in a Report from the Birmingham Infirmary, by Dr. Ogier Ward. The Report is published in the last volume of the Provincial Transactions.

11. *Successful Treatment of Lead-Colic.*

"Besides painters, &c., lead colic prevails among brass tap-makers, and glass polishers. The former mix 1-15th of lead with the metal, which 'sweats out' during the cooling, and is removed by the file and lathe: the latter make use of the oxide as a polishing powder. I rarely use opium internally, except to allay pain after the bowels have been well opened; and this is accomplished by turpentine and castor oil, or calomel and hyoscyamus, with a purgative by the mouth, and castor oil and turpentine in glysters, injected pretty forcibly to overcome the constriction, for such I believe to be the opposing cause, and not a paralysis of the intestine. These means, with opiate liniments to the abdomen, I have never known to fail of giving relief, where calomel and opium have been tried in vain."—*Ibid.*

12. *Treament of Pleurodynia.*

"The cases of simple pleurodynia were rare, but combined with amenorrhœa and dyspepsia they were very numerous. From having observed this pain in the side to be almost invariably excited or increased by taking food, and from its complication with dyspepsia or gastritis in so many instances, I cannot but conclude that it consists in an inflammation or irritation of the cardiac portion of the stomach. I cannot call to mind a single case of its occurrence in the right side. Except in highly hysterical and nervous patients I have had but little difficulty in removing it by leeches, blisters, and prussic acid or creosote. I have given bismuth frequently, but never with any benefit. When the pain has not been very obstinate, I have found the tartar emetic ointment of great service, applied over the seat of the pain or to the spine; for in these cases I have generally found the same pain is produced by tapping the spine, as by direct pressure over the part."—*Ibid.*

13. *Prevalence of Tape-worm in Birmingham.*

"The number of persons, chiefly women, affected with tape worm in this town is astonishing, the cause of which does not appear. I find the fern root very efficacious when, as is frequently the case, the patient cannot be induced to take the turpentine. One patient told me she had passed a worm that measured eighty yards!—*Ibid.*

